# Immunomodulatory functions of growth differentiation factor 15 in skin aging and inflammatory dermatoses

**DOI:** 10.3389/fimmu.2026.1902574

**Published:** 2026-07-31

**Authors:** Ruijun Wang, Haopeng Tan, Zhiqi Li, Dongmei Li, Yuanming Pan, Zhanbiao He

**Affiliations:** 1 Department of Neurosurgery, The Affiliated Hospital, Inner Mongolia Medical University, Hohhot, Inner Mongolia, China; 2 School of Medicine, Key Laboratory of Xinjiang Endemic and Ethnic Diseases, Shihezi University, Shihezi, Xinjiang, China; 3 Cancer Research Center, Beijing Chest Hospital, Capital Medical University/Beijing Tuberculosis and Thoracic Tumor Research Institute, Beijing, China

**Keywords:** biomarker, cutaneous immunomodulation, growth differentiation factor 15 (GDF15), inflammatory dermatoses, skin aging/photoaging

## Abstract

Growth differentiation factor 15 (GDF15) is a stress-responsive cytokine that has emerged as a pleiotropic regulator in skin biology, yet its context-dependent functions—ranging from protective to pathogenic—remain incompletely understood. This narrative review systematically synthesizes current evidence on the expression patterns, signaling mechanisms, and disease associations of GDF15 in cutaneous health and disease, with particular emphasis on melanogenesis, skin aging, photoaging, and immune-mediated dermatoses including psoriasis, systemic lupus erythematosus, and systemic sclerosis. We propose a four-parameter framework—inducing stimulus, responding cell type, exposure kinetics, and microenvironmental context—that reconciles the apparently contradictory effects of GDF15 across different skin compartments and disease phases. Mechanistically, GDF15 activates β-catenin/MITF/tyrosinase signaling in melanocytes to drive pigmentation, modulates fibroblast collagen synthesis via SMAD2/3 and SASP-associated pathways, and interfaces with the IL-23/Th17 and NF-κB axes in inflammatory skin diseases. Critically, we identify and discuss unresolved knowledge gaps, including the identity of the peripheral receptor(s) mediating GDF15’s cutaneous actions, the distinction between correlation and causation in human disease, and the therapeutic challenges posed by GDF15’s dual protective and deleterious roles. By integrating mechanistic insights with clinical observations and providing a critical assessment of current evidence levels, this review establishes GDF15 as a multi-node signaling hub in dermatology while offering a balanced roadmap for future translational research—emphasizing that the primary near-term value of GDF15 lies in biomarker development for disease monitoring and patient stratification, rather than in immediate therapeutic targeting.

## 1 Overview and research background of GDF15

This narrative review provides an integrated analysis of growth differentiation factor 15 (GDF15) in skin biology. A narrative format was chosen over a systematic review to allow detailed mechanistic discussion of pathway crosstalk and context-dependent phenomena—aspects that structured systematic reviews may underrepresent. While this approach offers greater interpretive depth, it carries inherent limitations: the literature search, though conducted systematically, cannot guarantee complete coverage of a rapidly expanding field, and a degree of selection bias is unavoidable. The emphasis throughout is on mechanistic insight and conceptual models to inform future investigation.

### 1.1 Literature search and study selection

This narrative review was conducted following established guidelines for transparent reporting of narrative reviews. A systematic literature search was performed in the following electronic databases: PubMed, Web of Science, and Scopus, covering the period from January 2000 to May 2026. The search strategy employed combinations of the following keywords and MeSH terms: *“growth differentiation factor 15”* OR *“GDF15”* OR *“MIC-1”* OR *“macrophage inhibitory cytokine 1”* AND *“skin”* OR *“cutaneous”* OR *“dermatology”* OR *“melanocyte”* OR *“fibroblast”* OR *“keratinocyte”* AND *“psoriasis”* OR *“sclerosis”* OR *“lupus”* OR *“pigmentation”* OR *“aging”* OR *“photoaging”* OR *“fibrosis”*.

Inclusion criteria were: (i) original research articles, reviews, and clinical studies published in peer-reviewed journals; (ii) studies investigating the role of GDF15 in skin biology, cutaneous diseases, or related mechanisms; (iii) articles written in English. Exclusion criteria were: (i) studies not directly related to GDF15 or skin; (ii) conference abstracts, case reports, and editorials; (iii) non-English publications. The screening process was conducted independently by two authors, with disagreements resolved by consensus. Given the narrative nature of this review, a formal PRISMA flow diagram was not included; instead, we adopted the SANRA (Scale for the Assessment of Narrative Review Articles) framework to ensure methodological quality and reporting transparency.

### 1.2 Biological attributes of GDF15 and the receptor question

GDF15, also known as macrophage inhibitory cytokine 1 (MIC-1), is a divergent member of the transforming growth factor-β (TGF-β) superfamily. It is synthesized as a precursor containing a pro-domain and a mature peptide; post-translational cleavage releases the biologically active dimeric form (1).

Although the canonical GDF15 receptor GFRAL is predominantly expressed in the brainstem, evidence from cell culture and ex vivo skin models demonstrates that GDF15 exerts direct effects on cutaneous cells—most notably, stimulating melanogenesis in melanocytes via β-catenin/MITF signaling (2). However, the receptor mechanism underlying these cutaneous effects remains incompletely characterized. Kim et al. (3) did not perform GFRAL-knockout or receptor-blockade experiments; therefore, it has not been formally established whether these effects are mediated through GFRAL expressed at low levels in peripheral tissues, through alternative receptor systems, or through receptor-independent mechanisms. The receptor for GDF15 in dermal fibroblasts also remains unknown. Accordingly, while the available data clearly demonstrate GDF15-driven cutaneous effects, claims of “GFRAL-independent” signaling in the skin should be considered provisional until definitive receptor studies are conducted.

#### 1.2.1 Upstream triggers and transcriptional control of GDF15 expression

Rather than viewing GDF15 primarily through the lens of its receptor, a more instructive starting point for understanding its cutaneous functions is the regulatory logic that governs its expression. GDF15 is a stress-responsive cytokine: its transcription is rapidly induced by a wide array of noxious stimuli, and the nature of the inducing stimulus—together with the responding cell type—largely dictates the subsequent biological outcome (4–7).

Stimulus-specific transcriptional programs. The *GDF15* promoter integrates signals from multiple stress-sensing transcription factors, creating a molecular decoder that translates distinct environmental inputs into graded GDF15 output: (i) Integrated Stress Response (ISR)/ATF4–CHOP axis: Activated by endoplasmic reticulum stress, amino acid deprivation, and mitochondrial dysfunction. In the skin, this pathway is engaged by UV-induced oxidative damage and by metabolic stress within hyperproliferative psoriatic epidermis, driving GDF15 upregulation as part of a cellular adaptation or survival program (8, 9). (ii) p53: Directly binds the *GDF15* promoter in response to DNA damage—particularly relevant to UV irradiation and photocarcinogenesis. This p53–GDF15 axis links acute genotoxic stress to downstream effects on melanocyte survival and pigmentation (10, 11). (iii) EGR1 (Early Growth Response 1): Mediates rapid, transient GDF15 induction following growth factor stimulation, mechanical stretch, and inflammatory cues. EGR1-dependent GDF15 expression is observed in wound healing and fibrotic remodeling (12, 13). (iv) NF-κB: Activated by pro-inflammatory cytokines (TNF-α, IL-1β) and Toll-like receptor signaling. In psoriatic skin, the TNF-α/NF-κB axis is a major driver of GDF15 elevation in both keratinocytes and dermal immune cells, placing GDF15 within the broader inflammatory network (14). (v) SASP-related regulators: In senescent fibroblasts—a hallmark of aged and photoaged skin—GDF15 is secreted as a core component of the senescence-associated secretory phenotype (SASP). This SASP-driven GDF15 contributes to paracrine signaling that disrupts tissue homeostasis, linking cellular senescence to pigmentary and matrix changes (15).

Cell-type specificity of GDF15 induction. Importantly, the relative contribution of these transcriptional programs varies across cutaneous cell types. In keratinocytes, NF-κB and p53 dominate in response to inflammation and UV, respectively. In fibroblasts, the ISR/ATF4 pathway and SASP regulators are prominent. In melanocytes, p53 and EGR1 are key mediators. This cell-type-specific regulatory landscape explains why the same systemic stimulus (e.g., UV exposure) can elicit quantitatively and qualitatively different GDF15 responses in different skin compartments.

#### 1.2.2 Downstream signaling pathways and cellular outputs

Once secreted, GDF15 engages cell-surface machinery to activate intracellular cascades. While the canonical GDF15 receptor GFRAL is primarily expressed in the brainstem and mediates systemic metabolic effects (16–18), the receptor(s) responsible for cutaneous GDF15 actions remain incompletely characterized. Current evidence points to alternative binding partners—including incompletely defined receptor complexes—that transduce signals in a cell-type-specific manner.

Despite this receptor uncertainty, several downstream effectors have been validated in skin-relevant contexts: (i) β-catenin: The most consistently documented cutaneous effector. In melanocytes, GDF15 activates β-catenin signaling, driving MITF and tyrosinase expression, thereby enhancing melanogenesis (3). This pathway is central to photoaging-associated hyperpigmentation. (ii) SMAD2/3: Observed in dermal fibroblasts, where GDF15 modulates collagen synthesis and matrix remodeling. The upstream receptor for this effect remains to be definitively identified, and the relationship between GDF15 and canonical TGF-β receptor/SMAD signaling requires further mechanistic clarification (19–22). (iii) AKT and ERK: Implicated in cell survival and proliferation responses across multiple cutaneous cell types, though the contextual determinants of their activation require further study (23–26). (iv) NF-κB: In immune cells, GDF15 can feed back to modulate NF-κB activity, creating a regulatory loop that either amplifies or dampens inflammation depending on the cytokine milieu (27).

A crucial distinction must be made: the downstream consequences of GDF15 signaling are not receptor-determined alone but are also shaped by the intracellular signaling network into which the GDF15 signal is integrated—a network that differs fundamentally between melanocytes, fibroblasts, keratinocytes, and immune cells.

#### 1.2.3 Deconstructing “context dependency”: specific parameters that determine GDF15 functional outcomes

Throughout this review, we invoke “context dependency” to explain the apparently contradictory functions of GDF15. To move beyond a vague generalization, we here explicitly define the four specific parameters that modulate GDF15 activity in skin (**Table 1**). This four-parameter framework—stimulus identity, cell type, exposure kinetics, and microenvironmental synergy—provides a structured lens for interpreting the divergent GDF15 phenotypes reported in the literature. Rather than representing intrinsic ambiguity, the contextual variability of GDF15 reflects its evolutionary role as a versatile stress sensor that tailors its output to the specific challenge and the specific tissue ecosystem in which that challenge occurs.

**Table 1:** Inducing stimuli, upstream transcription factors, and cutaneous consequences of GDF15 expression.

Parameter	Specific categories	Mechanistic consequence	Cutaneous example
Inducing stimulus (4, 11, 12, 14, 27–30)	UV radiation vs. inflammatory cytokines vs. metabolic/ER stress vs. mechanical stress	Engages distinct upstream TFs (p53 vs. NF-κB vs. ATF4/CHOP vs. EGR1), leading to differential GDF15 isoforms, secretion kinetics, and co-secreted factors	UV → p53 → GDF15 (pro-pigmentary); TNF-α → NF-κB → GDF15 (pro-inflammatory feedback)
Responding cell type (3, 21, 27, 31, 32)	Melanocyte vs. fibroblast vs. keratinocyte vs. immune cell	Differential expression of receptors, co-receptors, and downstream signaling competence; different basal transcriptional landscape	GDF15 → β-catenin/MITF in melanocytes (pigmentation); GDF15 → SMAD/COL in fibroblasts (fibrosis)
Duration and magnitude (3, 33)	Acute/transient vs. chronic/persistent elevation	Acute: adaptive/homeostatic (e.g., stress resistance, DNA repair); Chronic: maladaptive/pathological (e.g., sustained pigment deposition, matrix stiffening, immune dysregulation)	Acute UV-induced GDF15: photoprotective; chronic senescent fibroblast-derived GDF15: age-associated hyperpigmentation
Microenvironmental context (10, 14)	Presence of co-factors (cytokines, growth factors, ECM components)	Signaling cross-talk and pathway interference; GDF15 acts not in isolation but within a complex network	In inflamed skin, GDF15 plus IL-17 promotes keratinocyte hyperproliferation; GDF15 alone may not

#### 1.2.4 Framework for disease-specific analysis

Building on this framework, subsequent sections will analyze each cutaneous disease (pigmentary disorders, psoriasis, SLE, systemic sclerosis) by addressing three sequential questions: (i) What upstream stimulus/stimuli drive GDF15 elevation or suppression in this disease? (e.g., in psoriasis: TNF-α/NF-κB and IL-17/IL-22 pathways; in photoaging: UV-induced p53/ATF4 activation; in fibrosis: mechanical stress/EGR1 and TGF-β crosstalk); (ii) Through which cell type(s) and downstream pathways does GDF15 exert its effects? (e.g., melanocyte β-catenin/MITF, fibroblast SMAD/collagen, keratinocyte NF-κB); (iii) How do the duration, magnitude, and microenvironmental context shape whether GDF15 is protective or pathogenic in that condition?

This analytical structure directly addresses the reviewer’s concern that “context dependency” must be operationalized, not merely invoked.

The question of peripheral GDF15 receptors. Given the highly restricted expression of GFRAL to the brainstem, the receptor(s) through which GDF15 exerts its effects in peripheral tissues—including the skin—remain incompletely characterized and represent an active area of investigation (9, 16, 17).

Critically, despite initial hypotheses that GDF15 might signal through canonical TGF-β type I and type II receptors as a member of the TGF-β superfamily, multiple independent studies have failed to detect binding of mature GDF15 to any TGF-β type I or type II receptor—including comprehensive screens against more than 130 TGF-β family receptor combinations. A systematic receptor usage table further lists the type I receptor for GDF15 as “Unknown” and its type II receptor as TGFBR2 with “ND” (not determined), underscoring the absence of definitive evidence for canonical TGF-β receptor-mediated GDF15 signaling. These findings necessitate a cautious interpretation of mechanistic studies that attribute GDF15 effects to TGF-β receptor/SMAD pathways, as such observations may reflect indirect crosstalk or experimental artifacts rather than direct receptor–ligand interactions (34–40).

The high-affinity receptor for GDF15, GFRAL, was simultaneously identified by four independent research teams in 2017 (16, 17, 34, 35). This receptor belongs to the GDNF family α-like receptors and is predominantly expressed in the area postrema and nucleus tractus solitarius of the brainstem, with extremely limited expression in peripheral tissues (2). Consequently, the receptor mechanism(s) through which GDF15 exerts its effects in the periphery—including the skin—has remained an unresolved scientific question.

In cardiac studies, evidence indicates that GDF15 can induce Smad7 expression in fibroblasts via GFRAL, thereby inhibiting TGF-β/Smad signaling and exerting anti-fibrotic protective effects (31). This observation suggests that GFRAL may be expressed at very low levels in certain peripheral tissues, or alternatively, that GDF15 may signal through as-yet-unidentified receptor complexes. In the skin, multiple studies have confirmed functional effects of GDF15—for example, promoting pigmentation in melanocytes through the β-catenin/MITF pathway (7), and modulating collagen synthesis in fibroblasts (3)—yet the upstream receptor(s) mediating these cutaneous actions have not been definitively identified to date.

Furthermore, recent studies have suggested that GDF15 may also act through GFRAL-independent mechanisms (41). For instance, at cancer-relevant concentrations, GDF15 exhibits pro-angiogenic activity on endothelial cells, and pharmacological blockade of GFRAL does not attenuate this response, supporting the existence of alternative receptor systems. Meanwhile, although GDF15 is structurally a member of the TGF-β superfamily, systematic ligand-receptor binding studies have failed to detect specific binding of mature GDF15 to any TGF-β type I or type II receptor (35). The SMAD pathway activation reported in some early studies may therefore arise from experimental artifacts due to trace TGF-β contamination in recombinant GDF15 preparations (42).

Taken together, these receptor uncertainties warrant a cautious interpretation of mechanistic studies on GDF15 in cutaneous biology. At present, the peripheral effects of GDF15 under both normal physiological and various pathological conditions remain largely unknown, and its precise modes of action await systematic elucidation (40). In this review, we will therefore rigorously distinguish between experimentally validated downstream effects (e.g., GDF15-driven β-catenin/MITF in melanocytes and modulation of fibroblast collagen metabolism) and receptor mechanisms that require further confirmation, avoiding over-inference of direct receptor-ligand relationships.

#### 1.2.5 GDF15 and β-catenin signaling: cell-type-specific evidence and knowledge gaps

Among the downstream signaling pathways activated by GDF15 in cutaneous cells, β-catenin has emerged as a central mediator. However, the evidence supporting GDF15–β-catenin crosstalk varies considerably across cell types, and the molecular mechanisms through which GDF15 modulates β-catenin activity remain incompletely defined. Below, we summarize the current state of evidence by cell type, distinguishing between experimentally validated findings and hypothetical or indirect associations.

Melanocytes (validated). The most direct and best-characterized evidence for GDF15–β-catenin signaling comes from studies on melanocytes. Kim et al. (3) demonstrated that GDF15 derived from senescent fibroblasts stimulates melanogenesis in normal human melanocytes through upregulation of MITF (microphthalmia-associated transcription factor) and tyrosinase via β-catenin signaling. Specifically: (i) GDF15 treatment increased β-catenin protein levels and transcriptional activity in melanocytes. (ii) GDF15-stimulated pigmentation was significantly reduced in melanocytes co-cultured with GDF15-knockdown fibroblasts. (iii) The stimulatory effect of GDF15 on pigmentation was confirmed in ex vivo cultured skin and in reconstituted human skin samples.

The downstream targets of this pathway include MITF (the master transcriptional regulator of melanocyte development and function) and tyrosinase (the rate-limiting enzyme in melanin biosynthesis). The phenotypic outcome is enhanced melanin production and pigment deposition, linking GDF15–β-catenin signaling to physiological pigmentation and aging-associated hyperpigmentation (3).

Important caveat: The precise mechanism by which GDF15 activates β-catenin—whether through receptor-mediated phosphorylation, inhibition of GSK3β, or other intermediates—has not been fully elucidated. Furthermore, as discussed in Section 1.1.1, the upstream receptor mediating this effect remains unidentified.

Fibroblasts (limited/indirect evidence). In dermal fibroblasts, the relationship between GDF15 and β-catenin signaling is less clearly defined. Available evidence primarily concerns GDF15’s effects on collagen synthesis and extracellular matrix remodeling, with β-catenin involvement inferred rather than directly demonstrated. GDF15 has been shown to modulate the expression of ECM-related genes including COL1A1 and COL3A1 in normal human dermal fibroblasts (23). However, whether these effects are mediated through β-catenin, SMAD, or other pathways remains unclear. In other tissue contexts—such as kidney and cardiac fibroblasts—GDF15 has been reported to inhibit fibroblast survival and collagen production (21, 31), sometimes in association with TGF-β–SMAD pathway modulation (21), but direct evidence for GDF15-driven β-catenin signaling in dermal fibroblasts is currently lacking.

Keratinocytes (limited/indirect evidence). In keratinocytes, the relationship between GDF15 and β-catenin is even more tenuous. Studies in other cell types—particularly cancer models—have shown that GDF15 (also known as NAG-1) can inhibit β-catenin activity, for example by blocking EpCAM-induced β-catenin signaling and suppressing downstream targets such as c-myc and cyclin D1 (43). However, these observations come from colorectal and breast cancer contexts and have not been validated in cutaneous keratinocytes. Whether GDF15 exerts similar β-catenin-inhibitory effects in epidermal keratinocytes—and what the phenotypic consequences might be—remains an open question.

Summary and knowledge gaps. To date, the GDF15–β-catenin axis is best validated in melanocytes, where it drives MITF/tyrosinase-dependent melanogenesis. In fibroblasts and keratinocytes, the evidence is indirect or extrapolated from non-cutaneous contexts, and direct mechanistic studies are needed. Key unanswered questions include: (i) Through what molecular mechanism does GDF15 activate β-catenin in melanocytes? (ii) Does GDF15–β-catenin signaling operate in dermal fibroblasts, and if so, what are the phenotypic consequences? (iii) Are the β-catenin-modulatory effects of GDF15 in keratinocytes context-dependent (e.g., pro- vs. anti-proliferative)?

These knowledge gaps should guide future investigation and temper broad generalizations about GDF15 as a universal β-catenin modulator across all cutaneous cell types (**Table 2**).

**Table 2:** The level of evidence for GDF15 as a universal β-catenin modulator across all cutaneous cell types.

Cell type	Evidence for GDF15–β-catenin	Downstream targets	Phenotypic outcome	Evidence strength
Melanocytes (3)	Validated: GDF15 increases β-catenin protein/activity	MITF, tyrosinase	Melanogenesis, pigmentation	Strong (direct experimental evidence)
Fibroblasts (21, 22, 44, 45)	Limited/indirect: GDF15 modulates collagen genes; β-catenin involvement inferred	COL1A1, COL3A1 (putative)	ECM remodeling, fibrosis	Weak (direct evidence lacking)
Keratinocytes (3, 43)	Indirect/extrapolated: GDF15 inhibits β-catenin in cancer contexts	c-myc, cyclin D1 (in cancer)	Unknown in skin	Very weak (non-cutaneous evidence only)

### 1.3 Importance of GDF15 in skin health and disease

#### 1.3.1 Overview of GDF15 in pigmentation, aging, and immune regulation

Building on the regulatory framework established above, we now survey the major cutaneous domains in which GDF15 has been implicated. In each domain, the four parameters—stimulus, cell type, kinetics, and microenvironment—will be revisited to clarify why GDF15 exhibits divergent effects.

**Figure 1:**
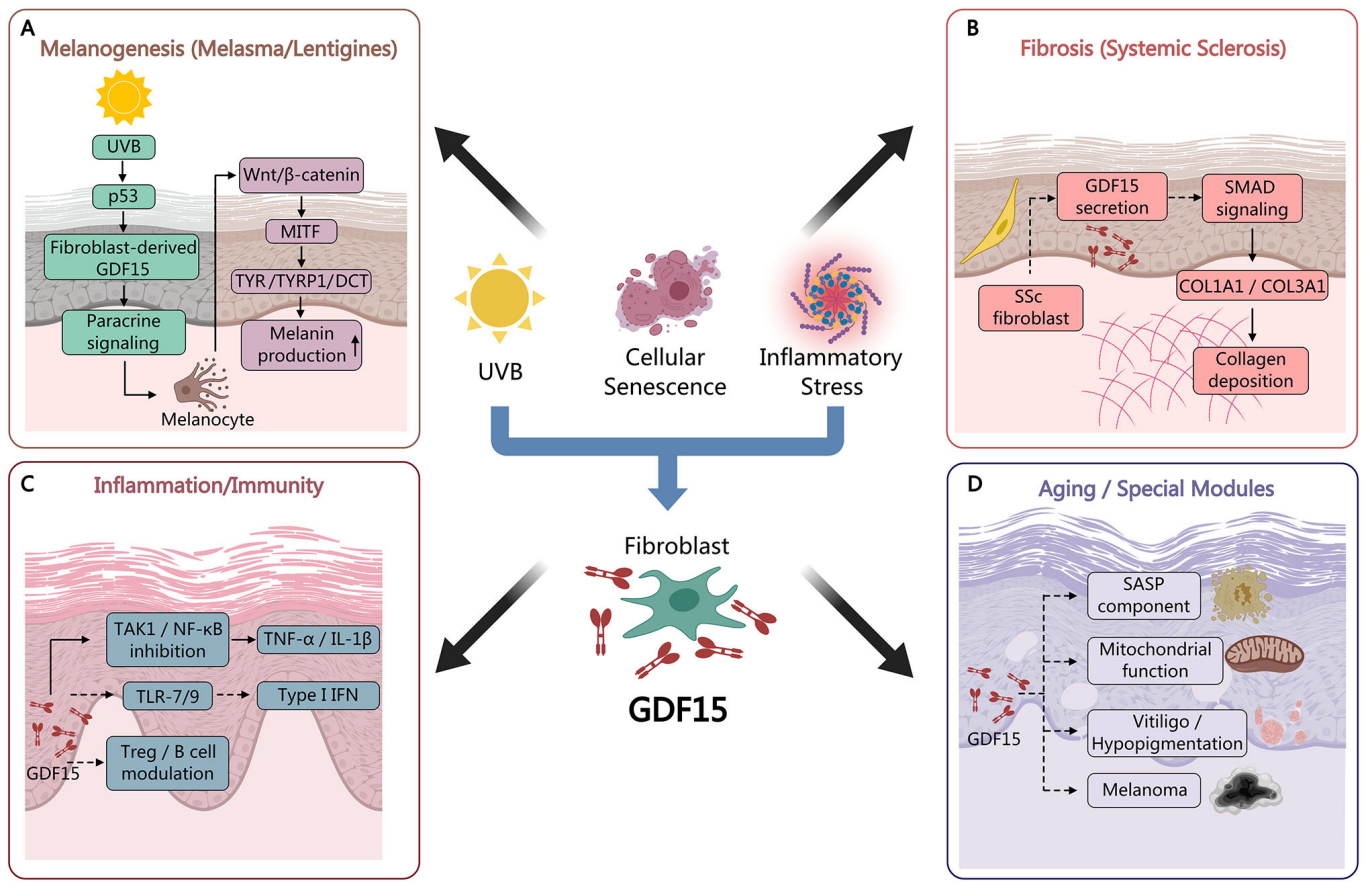
Schematic representation of GDF15-mediated signaling pathways in skin homeostasis and pathology. Environmental stressors such as UVB radiation, cellular senescence, and inflammatory stress converge on dermal fibroblasts to induce the secretion of GDF15 (Growth Differentiation Factor 15). Fibroblast-derived GDF15 subsequently acts as a central regulator of distinct pathological processes in the skin through paracrine and autocrine mechanisms. **(A)** Melanogenesis (Melasma/Lentigines): UVB exposure upregulates p53, promoting the secretion of GDF15 from fibroblasts. GDF15 acts in a paracrine manner on melanocytes. Concurrently, UVB activates the Wnt/β-catenin pathway, which upregulates MITF (Microphthalmia-associated Transcription Factor). This drives the expression of melanogenic enzymes (TYR, TYRP1, DCT), ultimately leading to enhanced melanin production, associated with hyperpigmentation disorders like melasma and lentigines. **(B)** Fibrosis (Systemic Sclerosis): In systemic sclerosis (SSc), aberrant SSc fibroblasts secrete GDF15. GDF15 activates SMAD signaling, which transcriptionally upregulates collagen type I (COL1A1) and collagen type III (COL3A1). This cascade promotes excessive collagen deposition, resulting in dermal fibrosis. **(C)** Inflammation/Immunity: GDF15 exerts immunomodulatory effects by inhibiting the TAK1/NF-κB pathway, thereby suppressing the production of pro-inflammatory cytokines such as TNF-α and IL-1β. Additionally, GDF15 modulates the TLR-7/9 pathway, affecting Type I Interferon (IFN) production, and regulates the activity and balance of T regulatory (Treg) and B cells, impacting cutaneous immune homeostasis. **(D)** Aging/Special Modules: In the context of skin aging, fibroblast-derived GDF15 is associated with the modulation of the Senescence-Associated Secretory Phenotype (SASP) and impacts mitochondrial function. Furthermore, dysregulation of GDF15 signaling is linked to pathological pigmentary disorders and malignancies, specifically involving vitiligo/hypopigmentation and melanoma.

GDF15 shows marked upregulation during melanogenesis, particularly under UV exposure and chronic inflammation (**Figure 1**). Senescent fibroblasts secrete GDF15, which stimulates melanocytes via β-catenin/MITF/tyrosinase pathway activation, leading to enhanced melanin production and pigment deposition. The receptor through which GDF15 acts on melanocytes—whether GFRAL or an alternative receptor—has not been definitively identified, as GFRAL-knockout studies in skin have not been reported. Throughout this review, we therefore refer to GDF15-mediated cutaneous effects without assuming receptor identity, and we explicitly distinguish between experimentally validated downstream signaling (e.g., β-catenin/MITF activation) and unresolved receptor mechanisms.

The effects of GDF15 on pigmentation are concentration-dependent: physiological levels support normal melanocyte function, whereas pathological elevations are associated with hyperpigmentation disorders (3, 28). This concentration-dependent behavior involves both Wnt/β-catenin and PKA/CREB pathways, consistent with a regulatory network rather than simple linear activation. The timing of GDF15 expression also matters: acute elevations may protect against UV damage, whereas chronic elevation appears to promote persistent pigmentary changes in aging and photoaged skin (10, 11, 33).

Beyond pigmentation, GDF15 affects multiple processes associated with skin aging. GDF15 modulates fibroblast function and extracellular matrix homeostasis through interactions with inflammatory mediators and metabolic regulators. These effects involve both direct regulation of collagen synthesis and indirect modulation of matrix metalloproteinase activity, contributing to the structural changes characteristic of aged skin (45). GDF15 also participates in immune regulation, affecting adaptive and innate responses, including T-cell differentiation, dendritic cell maturation, and macrophage polarization (1, 22, 26). Aberrant GDF15 signaling has been linked to immune-mediated skin disorders such as systemic lupus erythematosus and psoriasis; in these conditions.

#### 1.3.2 Main goals and scope of the review

This review addresses the expanding roles of GDF15 in dermatology, with attention to both fundamental mechanisms and clinical relevance. Topics covered include the molecular basis of pigmentation, mechanisms of skin aging, and immune dysfunction in cutaneous disease. Specific diseases—psoriasis, systemic lupus erythematosus, and systemic sclerosis—are discussed to illustrate how GDF15 contributes to cutaneous pathology (3, 14, 28, 46).

A central analytical goal across each disease section is to identify which upstream signals drive GDF15 elevation or suppression in that specific condition, rather than merely documenting changes in GDF15 levels. For example, in psoriasis, we consider whether GDF15 induction is driven by TNF-α/NF-κB signaling, IL-17/IL-22-mediated responses, or metabolic stress within lesional skin (14, 46). Similarly, in photoaging, UV-induced p53 and ATF4 activation are the upstream drivers (3, 28, 47). This stimulus-to-pathway-to-outcome approach aims to provide a mechanistic framework that can inform therapeutic targeting.

Our analysis indicates that GDF15 exerts bidirectional effects across several skin processes. In pigmentation, GDF15 can enhance melanogenesis via growth factor pathways while also modulating it through inflammatory signaling (3, 28, 47). In aging, GDF15 appears protective against acute stress yet may contribute to chronic degenerative changes (5, 15, 48). In immune regulation, GDF15 can promote either pro-inflammatory or anti-inflammatory responses, depending on cellular context and disease stage (14, 49, 50). We also consider therapeutic strategies targeting GDF15 pathways, including small molecule modulators, neutralizing antibodies, and receptor directed approaches (51–53). Developing such interventions will require attention to GDF15’s pleiotropic effects and the potential need for tissue specific or temporally controlled delivery. In sum, we present GDF15 as a factor of growing importance in the development of targeted therapies for skin disorders (28).

## 2 GDF15 and melanogenesis

### 2.1 Molecular mechanisms of melanogenesis and the role of GDF15

#### 2.1.1 MITF regulatory pathways and the roles of CREB and Wnt/β-catenin signaling

**Figure 2:**
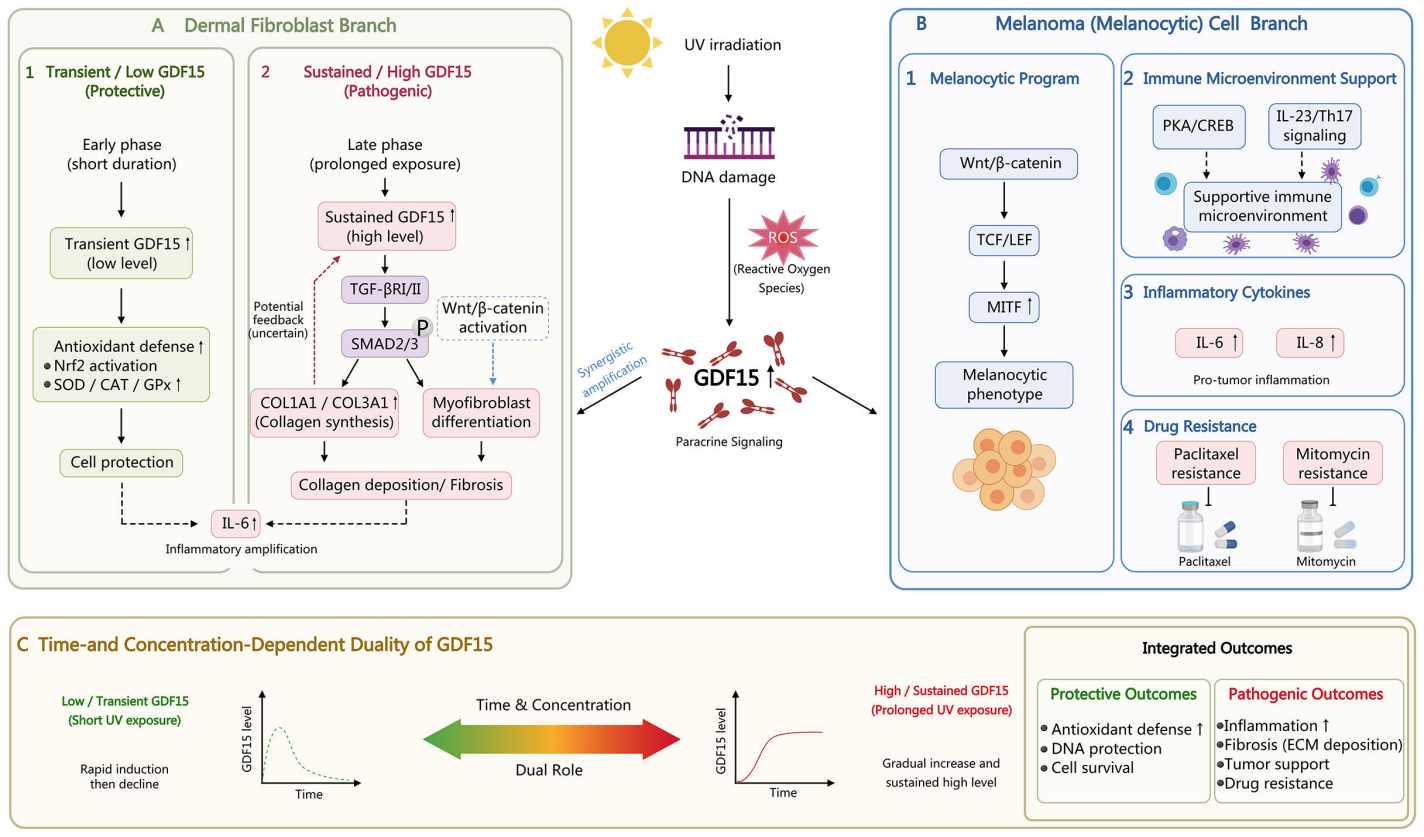
The time- and concentration-dependent duality of fibroblast-derived GDF15 in UV-irradiated skin and melanoma pathogenesis. UV irradiation induces DNA damage and Reactive Oxygen Species (ROS) in the skin, triggering the secretion of Growth Differentiation Factor 15 (GDF15). This schematic illustrates how GDF15 exerts distinct, concentration-dependent paracrine effects on dermal fibroblasts and melanoma cells, leading to dichotomous pathophysiological outcomes. **(A)** Dermal Fibroblast Branch: In fibroblasts, GDF15 exhibits a biphasic response depending on exposure duration. (i) Transient/Low GDF15 (Protective Phase): During the early phase (short UV exposure), transiently elevated GDF15 promotes antioxidant defense via the Nrf2 pathway, upregulating enzymes like SOD, CAT, and GPx, leading to cellular protection. (ii) Sustained/High GDF15 (Pathogenic Phase): During the late phase (prolonged UV exposure), sustained high levels of GDF15 activate the TGF-βRII/SMAD2/3 pathway and synergize with Wnt/β-catenin activation. This drives the expression of COL1A1/COL3A1, promotes myofibroblast differentiation, and induces excessive collagen deposition, resulting in dermal fibrosis. A potential feedback loop between GDF15 and IL-6 also contributes to inflammatory amplification. **(B)** Melanoma (Melanocytic) Cell Branch: GDF15 functions as a paracrine signal acting on melanoma cells, promoting tumor progression through multiple mechanisms: (i) Melanocytic Program: GDF15 activates the canonical Wnt/β-catenin → TCF/LEF pathway, which upregulates MITF, thereby reinforcing the melanocytic phenotype. (ii) Immune Microenvironment Support: GDF15 promotes PKA/CREB and IL-23/Th17 signaling cascades, fostering a supportive and immunosuppressive tumor microenvironment. (iii) Inflammatory Cytokines: GDF15 induces the secretion of pro-inflammatory cytokines, specifically IL-6 and IL-8, contributing to pro-tumor inflammation. (iv) Drug Resistance: GDF15 upregulation confers resistance to chemotherapeutic agents, notably enhancing resistance to both Paclitaxel and Mitomycin. **(C)** Time-and-Concentration-Dependent Duality of GDF15: The biological function of GDF15 is strictly determined by its spatiotemporal kinetics. Transiently low levels of GDF15 (rapid induction followed by decline) lead to protective outcomes (antioxidant defense, DNA protection, and increased cell survival). Conversely, sustained high levels of GDF15 induced by prolonged UV exposure lead to pathogenic outcomes, including aggravated inflammation, dermal fibrosis (ECM deposition), tumor microenvironment support, and drug resistance in melanoma.

Microphthalmia-associated transcription factor (MITF) is the master regulator of melanogenesis (54–56). It controls the expression of key melanogenic enzymes-tyrosinase (TYR), tyrosinase-related protein 1 (TYRP1), and tyrosinase-related protein 2 (TYRP2)-which are essential for melanin synthesis. MITF activity is governed by upstream signals, most notably cAMP response element-binding protein (CREB) and Wnt/β-catenin pathways. These pathways regulate melanocyte function and pigment production (57) (**Figures 1A**, **2B**).

CREB signaling is among the best-characterized regulators of melanogenesis. Following activation by protein kinase A (PKA), phosphorylated CREB enhances *MITF* transcription, which in turn upregulates melanin-biosynthesis enzymes including TYR (58). For example, vasoactive intestinal peptide (VIP) promotes melanogenesis through CREB activation in B16F10 melanoma cells, increasing tyrosinase activity and melanin content. Conversely, pharmacological inhibition of CREB-associated coactivators such as CRTC3 suppresses melanogenesis, underscoring the importance of this pathway in pigmentation (59, 60) (**Figure 2B**).

Wnt/β-catenin signaling also contributes to MITF regulation. Pathway activation leads to β-catenin nuclear translocation, where it forms complexes with TCF/LEF transcription factors and promotes *MITF* expression. Studies in ex vivo human skin and co-culture systems suggest that senescent fibroblast-derived GDF15 can enhance β-catenin activity in melanocytes under UV exposure (**Figure 2B**) (3, 60).

Co-culture and ex vivo studies now provide direct evidence that GDF15 upregulates MITF, with β-catenin signaling identified as the principal mediator. The net effect on melanogenesis is not fixed; it shifts with local cues and the activation state of neighboring cells (3). How this context dependence is governed, and how GDF15 coordinates with CREB-driven transcription, remain open questions.

#### 2.1.2 Regulation of pigment metabolism by GDF15 under UV-induced, inflammatory, and aging conditions

Melanogenesis responds to environmental triggers including ultraviolet (UV) radiation, inflammation, and aging. These stressors directly affect melanocyte function and pigment metabolism. GDF15 appears to integrate these environmental signals into melanocyte responses (3, 11, 47).

UV radiation is a potent inducer of melanogenesis, primarily through activation of cAMP signaling and upregulation of MITF, TYR, and related enzymes (3, 33, 55, 61). UV-induced fibroblast senescence leads to increased GDF15 secretion, which in turn amplifies melanocyte activity via β-catenin signaling. Ex vivo human skin models have confirmed that elevated GDF15 levels correlate with enhanced melanin accumulation under UV exposure (**Figure 1**).

The hypothesis that GDF15 might regulate melanogenesis through voltage-gated sodium channels (Nav1.7/1.8) remains entirely speculative, with no supporting experimental evidence in melanocytes or any other skin cells (62, 63). Such a mechanism, proposed on the basis of sodium-channel studies in other cell types, awaits experimental validation in skin.

During inflammation, cytokines released from neighboring cells can influence melanogenesis. Given its immunomodulatory effects in other tissues, GDF15 may modulate the relationship between inflammation and pigment synthesis (64). However, current evidence in skin is limited, and whether GDF15 promotes or suppresses melanogenic factors under inflammatory conditions requires clarification (3, 28, 33). The role of GDF15 in inflammatory pigmentation disorders is not well understood, though UV and cytokine studies point to a regulatory function that merits further investigation.

Aging complicates pigmentation regulation because cellular senescence alters melanocyte and fibroblast behavior (65). GDF15 levels increase significantly in aged skin fibroblasts, and this elevation correlates with pigmentation disorders including age spots and lentigines (3). The aged skin microenvironment, with elevated GDF15, promotes the upregulation of pigmentation genes including *MITF* and *TYR* (56, 60). Some experimental studies suggest that aged skin environments enriched with GDF15 can upregulate pigmentation-related genes (65). These findings indicate a potential contribution of GDF15 to age-related pigment alterations, but confirmation in large-scale, well-controlled studies is still needed (66). Targeted modulation of GDF15 may offer therapeutic possibilities for pigmentation abnormalities linked to aging and chronic UV exposure.

GDF15-mediated melanogenesis involves cross-talk among multiple signaling pathways. The interaction between PKA-CREB and Wnt/β-catenin pathways creates redundancy and feedback mechanisms that help maintain pigmentation homeostasis (3, 43, 61, 67–69). These feedback loops allow fine-tuning of melanogenic responses; GDF15 acts as a modulator that integrates environmental and cellular stress signals (**Figures 1A**, **2B**).

GDF15 modulates melanogenic signaling intensity through feedback mechanisms involving both β-catenin and CREB pathways (3). Sustained secretion of GDF15 by senescent fibroblasts may create a feed-forward loop that maintains elevated β-catenin activity in melanocytes (33). Under chronic oxidative stress, persistent elevation of GDF15 could disrupt these regulatory mechanisms, thereby contributing to pigmentary disorders. GDF15 has been shown to activate MAPK and NF-κB pathways in other biological contexts (70). Whether these non-canonical pathways directly mediate GDF15’s effects on pigmentation in human skin remains uncertain and represents an important direction for future research (3).

### 2.2 Pathological mechanisms of melanogenesis and clinical relevance of GDF15

#### 2.2.1 Bidirectional regulatory mechanisms of GDF15 in photoaging and related pigmentary disorders

Photoaging, resulting from chronic UV exposure, accelerates pigmentation irregularities through dysregulated melanocyte activity (3). Under these conditions, GDF15 appears to exert bidirectional control over pigmentation, both promoting and modulating melanogenic signaling (28).

In photoaged skin, senescent fibroblast-derived GDF15 enhances melanin production by upregulating MITF and tyrosinase via β-catenin signaling (3). Under inflammatory conditions associated with photoaging, GDF15 may dampen excessive activation of pigmentation pathways (14, 30, 33, 71, 72). This possibility has been suggested but awaits clinical verification.

Elevated GDF15 levels have been reported in pigmentary disorders such as melasma and solar lentigines. A clear quantitative correlation between GDF15 expression and lesion severity has not been established; prospective studies are needed. Whether targeting fibroblast-derived GDF15 can mitigate photoaging-associated hyperpigmentation remains to be tested.

#### 2.2.2 Therapeutic strategies developed based on GDF15 regulation mechanisms

The diverse functions of GDF15 in melanogenesis suggest therapeutic relevance for both hyperpigmentation and hypopigmentation disorders. Therapeutic strategies may involve either inhibition or enhancement of GDF15 signaling, depending on clinical context (3, 28, 73, 74).

In hyperpigmentation, GDF15 inhibitors could suppress β-catenin-driven hyperactivation of MITF and reduce excessive melanin synthesis (3). RNA-based and small-molecule approaches to reduce GDF15 secretion from senescent fibroblasts have been proposed (75). These approaches remain at early developmental stages and have not entered clinical trials. Combining GDF15 inhibitors with UV-blocking agents may yield synergistic effects.

In conditions such as vitiligo, where melanin synthesis is compromised, exogenous GDF15 supplementation or pathway agonists have been proposed to support melanogenesis (3, 28) (**Figure 1D**); however, these strategies remain untested. Precise modulation of GDF15 signaling-ideally with minimal off-target effects-could correct pigmentary imbalances while maintaining skin homeostasis (47). Realizing this potential will require collaboration across molecular biology, dermatology, and pharmacology.

## 3 GDF15 in skin aging

### 3.1 Dual regulatory roles of GDF15 in skin aging

#### 3.1.1 Core molecular networks of skin aging and key roles of GDF15

Skin aging arises from interactions between intrinsic and extrinsic factors (76–78). Intrinsic aging results from genetic and metabolic changes over time, whereas extrinsic aging primarily stems from environmental exposures, including ultraviolet (UV) radiation, pollution, and oxidative stress (79–81). In photoaging, senescent fibroblast-derived GDF15 activates β-catenin signaling in melanocytes, leading to MITF and tyrosinase upregulation and subsequent melanin overproduction. This GDF15–β-catenin–MITF axis represents the best-characterized mechanistic pathway linking GDF15 to cutaneous pathology.

Key molecular networks involved include TGF-β, MAPK, and oxidative stress signaling pathways, which regulate collagen synthesis, inflammatory responses, and apoptosis (81–83). These pathways are heavily influenced by reactive oxygen species (ROS), which accumulate with age and contribute to cellular damage and senescence (77, 79).

GDF15 participates in these networks and may function as either a protective or detrimental factor in skin aging (5, 15). As a member of the TGF-β superfamily, GDF15 influences fibroblast function, inflammatory signaling, and oxidative stress responses (4, 48, 84–86). GDF15 may downregulate inflammatory pathways by modulating cytokine secretion, thereby mitigating the effects of chronic inflammation on the skin extracellular matrix (ECM). Under prolonged UV exposure, GDF15 overexpression may exacerbate matrix degradation by upregulating matrix metalloproteinases (MMPs), promoting wrinkle formation and skin thinning (48) (**Figure 2A**). Limited in-skin evidence exists linking GDF15 to MMP expression; however, GDF15 has been reported to upregulate MMPs in tumor contexts and is co-expressed with MMP12 in senescent skin, raising the possibility that GDF15 may exacerbate ECM degradation in photoaged dermis under chronic elevation. The role of GDF15 in aging is context-dependent, influenced by timing, localization, and expression level rather than fixed thresholds.

#### 3.1.2 Regulation of fibroblast function, inflammatory factors, and oxidative stress by GDF15

Dermal fibroblasts maintain skin scaffolding by producing ECM proteins, including collagen and elastin (4, 48, 87). Senescence disrupts this homeostasis. GDF15 modulates fibroblast function by influencing ECM secretion and responses to ROS and inflammatory stimuli (15, 64, 88). It helps fibroblasts resist oxidative stress, potentially delaying ECM degradation.

Beyond fibroblast function, GDF15 regulates inflammatory markers (14, 89, 90). Aging skin often exhibits low-grade chronic inflammation, termed “inflammaging,” which accelerates skin aging (89). GDF15 has been implicated in controlling chronic inflammation by inhibiting the production of pro-inflammatory cytokines such as TNF-α and IL-1β (14, 27, 89). By suppressing these cytokines, GDF15 can reduce oxidative stress-related cellular damage in dermal fibroblasts (15, 48, 89). Under prolonged inflammatory or UV-damaged conditions, excessive GDF15 may promote maladaptive changes, including increased MMP activity (33, 48, 90–92) (**Figure 2B**).

Cholinergic signaling represents a purely theoretical mechanism that might influence fibroblast behavior (3, 15–17, 35, 48). However, any proposed interaction between GDF15 and cholinergic receptors in dermal fibroblasts remains entirely hypothetical without direct experimental validation (48, 93). While GDF15 has been hypothesized to interface with this pathway, direct evidence in human dermal fibroblasts is currently lacking. Any proposed “GDF15 → cholinergic receptor → ECM homeostasis” mechanism should be considered hypothetical pending future validation (48).

Acute, transient elevations of GDF15 may align with protective stress responses, promoting cell survival and temporary matrix stabilization (5, 94, 95). In contrast, chronic, sustained increases-as observed in photodamaged skin-are more frequently associated with prolonged inflammatory signaling and ECM degradation (3, 96, 97). This temporal switch remains to be validated in longitudinal human studies (98).

#### 3.1.3 GDF15-related mechanisms in pigment deposition during photoaging

Pigmentary alterations are characteristic of photoaged skin and arise from keratinocyte-melanocyte interactions as well as paracrine influences from fibroblasts. GDF15 regulates pigment deposition in photoaged skin by acting on the enzymatic cascade-TYR, TYRP1, and MITF-that governs melanin output (3, 99). UV radiation induces oxidative stress and DNA damage, which upregulate GDF15 expression alongside pro-melanogenic signaling proteins. This enhances melanin production as a protective response to UV damage, resulting in hyperpigmentation disorders such as solar lentigines and melasma.

GDF15 can also modulate melanocyte activity indirectly through effects on fibroblasts (3). Fibroblasts in UV-stressed environments release paracrine factors that influence melanocytes, but direct evidence linking these secretions specifically to GDF15 remains limited (3, 100, 101). Whether GDF15 drives the production of factors such as stem cell factor (SCF) or keratinocyte growth factor (KGF) *in vivo* remains to be confirmed (102). Dysregulated GDF15 expression in senescent or photoaged fibroblasts may drive uneven pigmentary changes and exacerbate age-related pigment deposition (28, 61).

### 3.2 Association between GDF15 and aging-associated pigment deposition

Aging fibroblasts acquire a senescence-associated secretory phenotype (SASP), secreting pro-inflammatory cytokines, growth factors, and proteases that collectively disrupt skin homeostasis (103). Among these factors, IL-6 and IL-8 have been associated with melanin overproduction and irregular pigmentation in aged skin. GDF15 and SASP cytokines interact in a regulatory loop (103, 104). The upstream arm is well documented: IL-6 and IL-8 from senescent fibroblasts induce *GDF15* transcription (10, 96, 105). The downstream arm-whether GDF15 in turn amplifies SASP secretion-is plausible but less firmly established, and its contribution to pigment dysregulation awaits direct testing (15) (**Figure 1C**).

GDF15 also interacts with the p53 pathway, which regulates both premature senescence and pigmentation (11, 28). This molecular crosstalk may connect fibroblast senescence to hyperpigmentation, though causal relationships require validation in human studies.

Fibroblast-melanocyte co-culture systems and patient-derived organotypic models represent valuable platforms for investigating the effects of GDF15 on pigment production and ECM remodeling (3, 45). Clinical translation of these findings remains at an early stage (28, 47). GDF15-targeted therapies, including inhibitors for hyperpigmentation and agonists for protective signaling, require rigorous preclinical evaluation before clinical application (3, 28, 33, 45, 48).

The biological effects of GDF15 in aging skin are context-dependent (3, 96). Transient GDF15 elevation may confer protection against acute stress, whereas chronic overexpression associated with senescence and UV damage promotes persistent pigmentary and structural alterations (3, 15, 48, 106). Interventions targeting GDF15 should account for timing, tissue localization, and inflammatory status; fixed expression thresholds remain unreliable given current knowledge gaps (45, 96).

## 4 GDF15 and major skin immune diseases

### 4.1 Role of GDF15 in psoriasis and systemic lupus erythematosus

#### 4.1.1 GDF15 and the IL-23/Th17 axis: disease-specific regulatory patterns

The IL-23/Th17 axis is a central inflammatory pathway in psoriasis and systemic lupus erythematosus (SLE) (107, 108). Although both diseases exhibit dysregulated IL-23/Th17 signaling, GDF15 appears to exert distinct modulatory effects in each condition (14, 109, 110) (**Figure 2B**).

GDF15 expression patterns in psoriasis remain controversial. Early studies reported elevated GDF15 levels in serum and lesional skin, with correlations to Psoriasis Area and Severity Index (PASI) scores (71, 72). Subsequent investigations have yielded contradictory results, with some reports indicating decreased GDF15 in psoriatic lesions compared with healthy skin (14). Whether GDF15 is elevated or reduced in psoriasis remains unresolved, possibly reflecting heterogeneity in patient populations or measurement methodologies. Consequently, no consensus exists regarding whether GDF15 promotes or limits psoriatic inflammation. These discrepancies account for the designation of “conflicting findings” (72).

In SLE, GDF15 levels track with disease phase-rising during flares and falling in remission (111). Murine data further indicate that GDF15-deficient mice show exaggerated lymphoproliferation and autoantibody titers, pointing to an active suppressive role. Whether this translates to human lupus remains to be determined (110).

In psoriatic lesions, IL-17 and IL-22 activate keratinocytes and induce CCL20 secretion, which recruits additional immune cells and may perpetuate inflammation. Whether GDF15 participates in this inflammatory loop, and in what capacity, is not yet known (14, 112, 113). Available evidence cannot distinguish whether GDF15 represents a failed compensatory mechanism or an active contributor to pathological inflammation (14, 72).

The mechanistic basis for these disease-specific effects remains incompletely understood. GDF15 may modulate IL-23/Th17 signaling through concentration-dependent mechanisms; however, without consensus on its expression levels in psoriasis, this hypothesis remains speculative (**Figure 2B**) (14, 114). Resolution of these uncertainties will require standardized measurement protocols, larger patient cohorts, and controlled experimental studies (14, 111).

#### 4.1.2 GDF15 effects on immune cell populations: mechanistic insights and clinical implications

GDF15 also modulates broader immune cell populations, with distinct effects observed in psoriasis and SLE.

In psoriatic lesions, elevated GDF15 correlates with enhanced dendritic cell activation and antigen presentation capacity, which may perpetuate chronic inflammation. GDF15 may also influence macrophage polarization, though whether this favors M1 or M2 phenotypes appears context-dependent (110).

In SLE, the relationship between GDF15 and immune cell function is more complex. During acute disease phases, elevated GDF15 may enhance regulatory T-cell function and IL-10 production, potentially reflecting an immune regulatory response (54). Chronic elevation, however, may paradoxically impair immune tolerance, promoting autoantibody production and tissue damage (110, 111).

The potential of GDF15 as a biomarker for disease monitoring has also been investigated. In psoriasis, serum GDF15 levels correlate positively with PASI scores, suggesting utility for tracking disease severity (71, 115). In SLE, the biphasic expression pattern of GDF15 may inform assessment of disease activity and treatment response, though standardized measurement protocols remain to be established (111, 116).

### 4.2 Clinical application prospects of GDF15 in immune regulation

#### 4.2.1 Context-dependent effects of GDF15 in autoimmune skin diseases

GDF15 exerts context-dependent effects in autoimmune skin diseases, differing markedly between acute and chronic phases. This contextual variability is particularly evident in psoriasis, where human clinical data and murine experimental evidence currently present an apparent paradox that requires careful reconciliation.

##### 4.2.1.1 Human clinical evidence: elevated GDF15 and correlation with disease severity

Multiple clinical studies have established that circulating GDF15 levels are significantly elevated in psoriasis patients compared with healthy controls. A 2021 study found that serum GDF15 levels in patients with psoriasis were significantly higher than those in the control group and correlated positively with Psoriasis Area and Severity Index (PASI) scores (115). This study determined a serum GDF15 cutoff value of >1498.5 pg/mL for predicting high PASI scores (115). A subsequent 2025 case-control study confirmed that median GDF15 levels were significantly higher in patients compared with controls and showed significant correlation with both disease duration and PASI scores (*P* < 0.001) (71). This study further proposed that GDF15 may act as a potential link between psoriasis and metabolic syndrome (71).

Biomarker classification. Based on the available cross-sectional and severity-correlative data, GDF15 is best classified as a monitoring biomarker—a marker that reflects current disease activity and correlates with clinical severity. The cross-sectional design of these studies does not support classification as a prognostic biomarker (which predicts future disease course or complications) or a predictive biomarker (which predicts response to specific therapies). Prospective longitudinal studies are required to determine whether baseline or serial GDF15 measurements can predict disease progression, treatment response, or the development of psoriatic comorbidities.

##### 4.2.1.2 Murine experimental evidence: a protective role for GDF15

In apparent contrast to the human correlative data, a 2022 study in an imiquimod-induced mouse model of psoriasis reported that GDF15 levels were reduced in psoriatic lesions, and GDF15-knockout animals exhibited a more severe inflammatory phenotype, characterized by increased neutrophil infiltration and abnormal keratinocyte proliferation (14). Mechanistically, GDF15 deficiency was associated with attenuated TAK1/NF-κB activation—a finding that, paradoxically, appears to worsen rather than ameliorate disease. This observation implies that endogenous GDF15 serves a protective, inflammation-limiting role in the acute setting.

##### 4.2.1.3 Reconciling the human–murine discrepancy

The apparent discrepancy between human clinical data (GDF15 elevated with disease severity) and murine knockout data (GDF15 protects against inflammation) can be reconciled through several considerations: (i) Systemic versus local concentrations. Human studies typically measure circulating serum GDF15, which may reflect systemic metabolic or inflammatory burden rather than local cutaneous levels. In the murine imiquimod model, GDF15 was assessed locally within lesional skin, where levels were reduced. It is plausible that chronic systemic inflammation drives sustained elevation of circulating GDF15 (as a metabolic stress response), while local GDF15 production within lesional skin may be relatively deficient, creating a permissive environment for unopposed inflammation. This systemic–local disconnect has been observed for other inflammatory mediators and warrants direct investigation in paired serum/skin samples from psoriasis patients (14) (**Figure 2A**). (ii) Acute versus chronic phases. The imiquimod model represents an acute, self-limited inflammatory response (typically 5–7 days), whereas human psoriasis is a chronic, relapsing disease with years to decades of disease duration. The function of GDF15 in the acute setting (protective, limiting inflammation) may differ fundamentally from its role in chronic disease, where sustained elevation may reflect maladaptive stress responses or contribute to fibrosis and metabolic complications (71). This temporal distinction is consistent with the broader theme of GDF15’s context-dependent actions discussed throughout this review. (iii) Species differences. While GDF15 is highly conserved, subtle differences in receptor pharmacology, downstream signaling, and immune system organization between mice and humans cannot be excluded as contributing factors. (iiii) Comorbidity burden. Human psoriasis patients frequently have comorbid metabolic syndrome, cardiovascular disease, and obesity—conditions that themselves drive GDF15 elevation. The elevated GDF15 observed in psoriasis patients may partly reflect these comorbidities rather than psoriasis perse. This possibility is supported by the study linking GDF15 to metabolic syndrome in psoriatic patients (71). Murine models typically lack these comorbidities, potentially explaining the more uniform picture seen in animal experiments.

##### 4.2.1.4 A unified model: GDF15 as a context-dependent stress sensor in psoriasis

We propose a unified, testable model that accommodates both human and murine findings: In acute inflammation (imiquimod model), endogenous GDF15 serves a protective, inflammation-limiting function via TAK1/NF-κB regulation. In chronic human psoriasis—often accompanied by systemic metabolic stress—sustained GDF15 elevation reflects a maladaptive response to combined inflammatory and metabolic burden, serving as a readout of disease and comorbidity severity rather than a primary pathogenic driver (**Figure 2A**).

This model positions GDF15 as a stress sensor that adapts its role to the duration and systemic context of the inflammatory challenge.

#### 4.2.2 Clinical utility and limitations

Based on the current evidence, GDF15 may have clinical utility as follows (**Table 3**).

**Table 3:** Clinical utility and limitations of GDF15.

Biomarker class	Supported?	Evidence basis
Monitoring	Yes	Correlates with PASI, disease duration (71, 115)
Risk	Possible (needs validation)	Associates with metabolic comorbidities (71)
Diagnostic	Insufficient	Not validated as a stand-alone diagnostic marker
Prognostic	Not supported	Cross-sectional data cannot predict future outcomes
Predictive	Not supported	No data on treatment response prediction

Key limitations: that must be acknowledged: (i) All human studies to date are cross-sectional, preventing assessment of whether GDF15 changes precede or follow changes in disease activity. (ii) The specificity of GDF15 elevation for psoriasis is poor—GDF15 is elevated in numerous inflammatory, metabolic, and neoplastic conditions. (iii) The optimal cut-off value (>1498.5 pg/mL) for high PASI prediction requires validation in independent cohorts. (iiii) Whether GDF15 measurement provides added value beyond existing clinical assessments (PASI, inflammatory markers) has not been established.

Implications for Therapeutic Development: The protective function of GDF15 in acute murine psoriasis suggests that GDF15 augmentation could theoretically have therapeutic benefit in early or acute-phase disease. Conversely, the association of chronically elevated GDF15 with disease severity in human psoriasis raises the question of whether sustained GDF15 elevation contributes to maladaptive outcomes (e.g., fibrosis, metabolic dysregulation) that might warrant GDF15 reduction or signaling modulation. These opposing therapeutic possibilities underscore the critical need for phase-specific and context-specific approaches to GDF15-targeted interventions in psoriasis.

GDF15 in SLE: A Phase-Dependent Picture. In systemic lupus erythematosus (SLE), the evidence also points to phase-dependent effects. During disease flares, GDF15 appears to exert a braking effect on autoimmunity (110); knockout studies have shown worsened lymphoproliferation and humoral responses in murine lupus models (109, 110). However, the effects of chronic GDF15 elevation in SLE remain less well characterized, and whether sustained elevation eventually erodes tolerance or contributes to tissue damage remains speculative. This mirrors the psoriasis paradigm: acute-phase protective function versus chronic-phase uncertain consequences. The mechanistic basis for these phase-dependent effects likely involves differential engagement of signaling pathways (NF-κB, β-catenin, SMAD) across cell types and temporal stages, though their relative contributions require systematic experimental elucidation.

The protective function of GDF15 in acute murine psoriasis raises a theoretical possibility that GDF15 augmentation could have therapeutic benefit in early disease (110). However, this remains speculative, and the contrasting association of chronically elevated GDF15 with human disease severity suggests that any therapeutic strategy would require precise phase-specific modulation—a level of control not currently achievable with existing tools.

#### 4.2.3 Therapeutic considerations and future research directions

Current biologics targeting IL-17, IL-23, or interferon pathways have shown efficacy in psoriasis and SLE, yet additional therapeutic options remain needed (72).

GDF15-targeted interventions could complement existing therapies, provided several key challenges are addressed. The context-dependent effects of GDF15 suggest that therapeutic strategies will require tailoring to specific diseases and disease phases. In psoriasis, reducing GDF15 activity during chronic phases may resolve persistent inflammation. In SLE, enhancing GDF15’s regulatory functions during acute flares may prove beneficial (14, 110, 118).

Clinical translation faces significant challenges. GDF15’s pleiotropic functions and involvement in metabolic regulation raise concerns regarding off-target effects. Tissue-specific delivery of GDF15 modulators presents an unresolved technical challenge (119, 120). Dr. Berg, a cardiologist and assistant professor of medicine at Brigham and Women’s Hospital, Harvard Medical School, stated that the risk increased by more than twofold in the side effects of worsening heart failure (HF) events. Berg and his colleagues conducted an international, multicenter, proof-of-concept study named GARDEN-TIMI 74 to investigate whether ponsegromab, building on its success in cancer treatment, could lower GDF-15 levels in a HF population. Elevated circulating levels of this well-established biomarker are associated with increased HF symptom burden, worse physical dysfunction, and a higher risk of adverse HF outcomes. Through 22 weeks, the cumulative incidence of the composite endpoint of cardiovascular death and worsening heart failure was 19.8% in the ponsegromab group versus 11.6% in the placebo group for the composite endpoint (hazard ratio HR = 2.24; 90% confidence interval (CI): 1.43-3.50). This result was driven primarily by worsening HF events, which were reported in 19.3% of the treatment group compared with 9.1% of the placebo group for worsening HF alone (HR = 2.84; 90% CI: 1.74-4.64) (120).

Key research priorities include: standardized GDF15 assays across disease states, longitudinal studies of GDF15 temporal dynamics, and controlled trials evaluating GDF15-targeted interventions. Combination approaches integrating GDF15 modulation with existing therapies may yield synergistic benefits while minimizing risks. GDF15-based therapeutics remain experimental and require extensive preclinical validation (14, 121).

## 5 GDF15 in fibrosis and systemic sclerosis

Systemic sclerosis (SSc) is characterized by excessive extracellular matrix (ECM) deposition, systemic inflammation, and vascular abnormalities; collagen overproduction is a central pathogenic feature. Elevated GDF15 levels have been documented in SSc patients and correlate with the degree of skin sclerosis and the intensity of pulmonary fibrosis, suggesting a clinical association between GDF15 and fibrotic severity. However, the precise molecular mechanisms through which GDF15 contributes to fibrotic responses—and whether these effects are direct or indirect—remain subjects of active investigation (122) (**Figure 1B**).

In systemic sclerosis, Wnt/β-catenin signaling is hyperactivated and contributes to fibrotic responses (123). While GDF15 is elevated in SSc and correlates with disease severity, whether GDF15 directly modulates β-catenin activity in SSc fibroblasts has not been established (7). The fibrotic effects of GDF15 in dermal fibroblasts—including COL1A1 and COL3A1 upregulation—may involve pathways distinct from β-catenin, and this remains an area requiring mechanistic clarification (**Figure 1B**).

### 5.1 GDF15 and fibroblast activation: observed effects and mechanistic uncertainty

Experimental studies have demonstrated that GDF15 exposure influences fibroblast behavior, including effects on proliferation and the transcription of collagen genes (COL1A1 and COL3A1). In some experimental systems, GDF15 has been reported to increase SMAD2/3 phosphorylation, leading to the hypothesis that GDF15 may modulate fibrotic responses through pathways overlapping with canonical TGF-β signaling (87, 124).

However, several critical caveats must be emphasized. First, as discussed in Section 1.1.1, mature GDF15 does not bind directly to TGF-β type I or type II receptors. Therefore, any observed effects of GDF15 on SMAD2/3 phosphorylation cannot be attributed to direct GDF15–TGF-β receptor interaction (125). Instead, such effects—if reproducible—likely reflect indirect mechanisms, such as: (i) GDF15-induced expression of TGF-β ligands or activators; (ii) Cross-talk between GDF15-activated signaling cascades (e.g., via β-catenin, AKT, or ERK) and the SMAD pathway; (iii) GDF15-mediated modulation of the cellular microenvironment that secondarily influences TGF-β signaling.

Second, the receptor through which GDF15 acts on fibroblasts remains unknown. While GFRAL is the definitive receptor for systemic GDF15 effects, its expression in dermal fibroblasts has not been convincingly demonstrated, and alternative receptor candidates have not been validated (17, 126).

Third, the directionality of GDF15 effects on fibrosis appears context-dependent and may differ across tissues and experimental conditions. In the liver, for example, GDF15 has been reported to attenuate chemically induced fibrosis by inhibiting the TGF-β1/SMAD3 pathway via AMPK activation (125). In the heart, GDF15 induces Smad7 through GFRAL, which may exert anti-fibrotic effects (31). These observations suggest that GDF15’s role in fibrosis is not unidirectional but rather depends on tissue type, cellular context, and the specific pathological milieu.

### 5.2 GDF15 in systemic sclerosis: clinical associations and mechanistic gaps

In SSc, elevated circulating GDF15 levels have been consistently observed and correlate with disease severity (122). This clinical association raises the possibility that GDF15 may serve as a biomarker of fibrotic activity in SSc, though whether this reflects a causal role in pathogenesis or represents an epiphenomenon of tissue stress and inflammation remains to be determined (28, 127, 128).

Experimental evidence on the functional role of GDF15 in SSc fibrosis is limited and sometimes conflicting. Some studies report that GDF15 silencing or inhibition in fibroblasts reduces collagen synthesis in experimental models, suggesting a pro-fibrotic contribution (40, 45, 123) (**Figure 1B**). However, the mechanistic basis of this effect—whether direct or indirect, receptor-mediated or not—has not been rigorously established. Other studies have noted that GDF15 can interact with inflammatory cytokines such as IL-6 in SSc fibroblasts, with preliminary evidence suggesting feedback mechanisms that influence ECM deposition.

### 5.3 GDF15–TGF-β crosstalk: a framework for interpretation

Rather than modeling GDF15 as a direct activator of TGF-β receptor/SMAD signaling—a model inconsistent with receptor binding data—we propose that GDF15 and TGF-β pathways interact at the network level through several potential mechanisms (21): (i) Indirect modulation: GDF15 may regulate the expression or activity of TGF-β ligands, receptors, or downstream signaling components, thereby indirectly influencing TGF-β pathway output. (ii) Pathway convergence: GDF15-activated signaling cascades (e.g., those involving β-catenin, AKT, or AMPK) may converge on shared transcriptional targets with TGF-β/SMAD signaling, producing additive or synergistic effects on collagen gene expression without direct receptor-level interaction (125). (iii) Context-dependent antagonism: In certain contexts, GDF15 may activate pathways (e.g., AMPK) that negatively regulate TGF-β/SMAD signaling, as observed in the liver, potentially explaining reports of anti-fibrotic GDF15 effects (129). (iv) Microenvironmental modulation: GDF15 may alter the inflammatory or metabolic milieu of the fibrotic niche, secondarily influencing TGF-β activity and fibroblast behavior (40).

These network-level interactions are fundamentally distinct from direct receptor-mediated signaling and should be described as such. The theoretical framework proposing that the TGF-β:GDF15 ratio may predict fibrosis progression—while hypothetical and requiring longitudinal validation—captures the notion that the balance between these two pathways, rather than the activity of either alone, may determine fibrotic outcomes.

### 5.4 Therapeutic implications and future directions

The clinical association between GDF15 and SSc severity, together with experimental evidence that GDF15 modulation affects fibroblast function, suggests that GDF15 pathways may offer therapeutic opportunities. However, several fundamental questions must be addressed before GDF15 can be considered a viable anti-fibrotic target: (i) What is the receptor for GDF15 in dermal fibroblasts? Identifying the receptor is essential for understanding mechanism and for developing receptor-specific interventions (40, 128). (ii) Is GDF15 elevation in SSc causal or reactive? Distinguishing between driver and bystander status is critical for therapeutic prioritization (123, 128). (iii) How can the context-dependent pro- versus anti-fibrotic effects of GDF15 be reconciled and therapeutically exploited (40, 129)?

Until these questions are answered, claims about GDF15-directed anti-fibrotic therapy remain speculative. The manipulation of GDF15–SASP interactions represents one potential avenue, but this approach remains experimental and requires rigorous validation.

The clinical association between GDF15 and SSc severity, together with experimental evidence that GDF15 modulation affects fibroblast function, provides a rationale for further investigation but does not constitute evidence that GDF15 is a viable therapeutic target (122, 130). The fundamental questions identified above—receptor identity, causal versus reactive status, and context-specific mechanisms—must be addressed before any therapeutic development can proceed.

### 5.5 Targeted applications of GDF15 in pathophysiological interventions

#### 5.5.1 SASP-GDF15 feedback network in autoimmune fibrosis

The senescence-associated secretory phenotype (SASP) represents a pro-inflammatory cascade linking cellular senescence to autoimmune fibrosis in SSc. Key SASP components-IL-1β, IL-6, and TNF-α-activate fibroblasts and drive collagen overproduction, creating a persistent inflammatory microenvironment that accelerates fibrotic progression. GDF15 integrates into this network as both a downstream effector and an upstream regulator (96, 106, 131).

Current models propose a self-reinforcing cycle: SASP cytokines upregulate *GDF15* expression, which in turn amplifies SASP activation and cytokine release. This positive feedback loop appears to sustain chronic fibroblast activation and excessive ECM deposition, driving progressive fibrosis. While this framework offers conceptual clarity, clinical validation through patient-derived data and longitudinal cohort studies remains necessary (96, 123, 132).

Experimental studies have tested interventions targeting this axis. GDF15 inhibitors and SASP modulators reduce ECM deposition in preclinical models, though these results remain preliminary. The mechanistic links between GDF15 and SASP require further clarification, and whether disrupting this loop can effectively halt disease progression has not yet been established (40, 45, 133).

#### 5.5.2 Therapeutic considerations and early-stage interventions

GDF15 has attracted interest as a potential therapeutic target for SSc. Monoclonal antibodies directed against GDF15 are being developed to block its role in SMAD and SASP signaling cascades, aiming to reduce fibroblast activation and limit ECM deposition (**Figure 1B**) (40, 45, 123).

Small-molecule inhibitors targeting GDF15 or its receptors are also under investigation. The therapeutic concept involves interrupting GDF15-receptor interactions to modulate downstream pro-fibrotic pathways. Combining such inhibitors with anti-TGF-β agents may yield synergistic benefits, though this hypothesis requires validation through rigorous preclinical and clinical studies (134).

Gene-editing techniques such as CRISPR-Cas9 have been proposed for direct modulation of *GDF15* expression. While preclinical data suggest potential normalization of collagen production, these technologies remain highly experimental and face considerable safety and efficacy hurdles before clinical consideration.

Early therapeutic intervention appears critical. The optimal treatment window likely occurs during the inflammatory-to-fibrotic transition, before extensive structural remodeling becomes irreversible. Defining precise criteria for early intervention will require prospective longitudinal clinical studies (135, 136).

### 5.6 Mechanistic conclusion

GDF15 exhibits dual and context-dependent roles in SSc pathogenesis (28). Its interactions with TGF-β and SASP signaling form a self-reinforcing network that drives SMAD cascade activation and sustains a pro-inflammatory microenvironment, promoting persistent fibroblast activation and excessive ECM deposition (40, 123, 137). This dynamic contributes to irreversible tissue fibrosis. However, under certain conditions, GDF15 may exert counter-regulatory effects on TGF-β signaling, potentially acting as a brake on excessive fibrotic progression (**Figure 1B**) (21, 125).

The “SASP-GDF15-TGF-β” axis can be conceptualized as a concentration- and context-dependent regulatory system. The TGF-β:GDF15 ratio has been proposed as a theoretical indicator for predicting disease trajectory, with higher ratios suggesting transition from a reversible, inflammation-driven stage to an irreversible, structural fibrotic phase (138, 139).

This model suggests that the optimal therapeutic window occurs early, during the transition between inflammatory activity and established fibrosis (46). Future research, particularly prospective clinical and histopathological studies, will be essential to validate this hypothesis and guide precision treatment strategies for SSc (**Table 4**) (135, 139, 140).

**Table 4:** Context-dependent effects of GDF15 in skin biology.

Diseases	Expression pattern	Reported effect of GDF15	Pathways/Mechanisms	Notes & clinical implication
Physiological baseline (3, 114)	Low, barely detectable in most somatic tissues	Limited evidence for direct physiological role; promotes melanogenesis via β-catenin/MITF when present	Wnt/β-catenin → MITF (established); cAMP/PKA–CREB (speculative, requires validation)	Constitutive expression is low in most tissues; GDF15 is primarily a stress-induced cytokine induced under pathological or injury conditions
Acute UV stress (3, 11, 28, 33)	Transiently elevated (e.g., 2h post-UVB)	Protective: promotes melanogenesis via MITF/tyrosinase upregulation; secreted as an inflammatory cytokine in UVB-exposed skin cells	Established: p53-responsive transcriptional upregulation; Melanogenesis: β-catenin → MITF/tyrosinase activation (*not* CREB); Stress-responsive: MAPK signaling context	Short-term adaptive response; transient elevation represents p53-mediated stress response; GDF15 is a UVB-inducible cytokine with potential photoprotective implications.
Chronic UV exposure/Photoaging (3, 15, 106)	Persistently elevated in senescent fibroblasts	Pathological: drives age-related hyperpigmentation (solar lentigines) via melanogenesis induction; indirectly associated with ECM remodeling/MMP upregulation as part of the broader SASP milieu.	Direct (GDF15-specific): β-catenin feed-forward loop → MITF/tyrosinase activation; Background (SASP-associated): NF-κB signaling; MMP-mediated ECM degradation.	Chronic elevation correlates with dyspigmentation and solar lentigines formation; concurrent SASP-driven structural changes (wrinkles, elastosis) occur in the same photoaged microenvironment, linking senescent fibroblasts to pigmentary imbalance
Inflammation (cytokine-rich milieu) (14, 71, 86, 90)	Variable; may be elevated (serum) or reduced (tissue, e.g., psoriasis epidermis)	Bidirectional: suppresses pro-inflammatory cytokines (TNF-α, IL-1β) via NF-κB inhibition; GDF15 deficiency aggravates skin inflammation and promotes neutrophil infiltration in psoriasis models.	Anti-inflammatory: TAK1/NF-κB inhibition; IKK/IκBα-mediated NF-κB suppression; Immunomodulatory: Treg function modulation; Notch4-GDF15 axis in regulatory T cells.	Context-dependent: elevated serum levels may represent compensatory anti-inflammatory response to tissue stress; GDF15 deficiency promotes inflammation; therapeutic potential in psoriasis and other inflammatory diseases.
Systemic Lupus Erythematosus (110, 111, 116, 126, 135)	Elevated during flares; associated with disease activity	Immunosuppressive: suppresses lymphoproliferation and humoral autoimmunity; inhibits TLR-7/9-driven type I interferon signaling; reduces pro-inflammatory cytokines and autoantibody production in lupus models	Established: suppression of lymphocyte proliferation and activation; negative regulation of TLR-7/TLR-9 → type I IFN signaling;Associated: Treg modulation (emerging evidence); B cell/plasma cell regulation	Serum GDF-15 is overexpressed in SLE patients and correlates with disease activity; potential biomarker for disease activity and therapeutic target; GDF-15 administration ameliorates lupus-like disease in murine models; timing relative to disea
Systemic Sclerosis (28, 122, 123, 127, 128, 130)	Elevated in activated fibroblasts and serum of SSc patients; associated with disease severity	Fibrosis-associated: correlates with extent of skin sclerosis and pulmonary fibrosis severity; GDF15 is a TGF-β superfamily member implicated in fibrosis development;Context-dependent: may modulate SMAD-dependent and -independent TGF-β signaling; participates in fibrosis initiation but is not indispensable for fibrosis progression *in vivo*	Established: TGF-β superfamily member; SMAD-dependent and -independent TGF-β signaling modulation;Associated: SASP component (GDF15 is a core senescent cell secretory factor); IL-6/CCL2 co-regulation in fibroblasts; mitochondrial stress response marker	Serum GDF-15 is significantly elevated in SSc patients and correlates with diffuse cutaneous SSc, lung impairment, and disease activity; potential biomarker for disease progression and therapeutic target; GDF15 expression is induced during fibrosis development and correlates with lung function impairment
Aging skin/senescence (3, 15, 90, 142)	Chronically elevated in senescent fibroblasts and photoaged skin	Pro-pigmentary: drives age-related hyperpigmentation (senile lentigines/solar lentigines) via melanogenesis induction; Homeostatic: maintains mitochondrial function and delays cellular senescence onset	Direct (GDF15-specific): β-catenin → MITF/tyrosinase activation (*not* p53–MITF–TYR); Associated: SASP component (GDF15 is a core senescent cell secretory factor); GDF15 mediates stress-induced senescence via mitochondrial dysfunction pathways	Senescent fibroblast-derived GDF15 links aging dermal microenvironment to pigmentary imbalance; GDF15 serves as a biomarker for aging and cellular senescence; GDF15 function is modulated by age and cellular context, suggesting context-dependent rather than threshold-driven effects

## 6 Integrated perspective: cross-disease regulatory networks of GDF15 in skin

While the preceding sections have examined the roles of GDF15 in pigmentation, aging, inflammation, and fibrosis as discrete domains, these processes are not biologically isolated. In skin—a complex, multi-compartmental organ—GDF15 functions as a hub connecting multiple pathological and physiological states. Understanding these cross-domain interactions is essential for grasping the overarching biological landscape of GDF15 in cutaneous biology (3, 5, 14, 15, 28, 71) (**Figure 1**).

### 6.1 The inflammation–pigmentation axis: linking psoriatic inflammation to melanocyte function

Among the most biologically relevant cross-disease interactions is the connection between skin inflammation (exemplified by psoriasis) and pigmentary changes (melanogenesis). This link is clinically evident: psoriasis patients frequently exhibit post-inflammatory hyperpigmentation or hypopigmentation in resolved lesions, but the molecular bridge between these processes has been incompletely understood (**Figure 1C**).

Paradoxical downregulation of GDF15 in psoriatic inflammation. In psoriatic skin, the inflammatory milieu is characterized by elevated levels of TNF-α, IL-17, and IL-22, which activate NF-κB and STAT3 signaling in keratinocytes and dermal immune cells. However, contrary to what might be expected, GDF15 expression is decreased in the epidermis of patients with psoriasis and in imiquimod-induced psoriasis-like mouse models. Mechanistically, TNF-α suppresses GDF15 expression in keratinocytes by inhibiting the protein level of the transcription factor GATA2. This downregulation is functionally significant, as GDF15 deficiency aggravates the development of psoriatic lesions, evidenced by more severe skin inflammation in Gdf15-knockout mice (14) (**Figure 1C**).

Bidirectional consequences of GDF15 insufficiency. The reduction of GDF15 in the psoriatic milieu has dual effects that may link inflammation to tissue pathology: (i) Loss of anti-inflammatory feedback (within the immune compartment): GDF15 normally limits the synthesis of keratinocyte cytokines and chemokines by inhibiting TAK1/NF-κB activation (14). GDF15 also directly inhibits neutrophil adhesion and migration by suppressing activation of the small GTPase Rap1. Thus, GDF15 serves a protective, homeostatic role that limits excessive inflammation, and its downregulation in psoriasis removes this brake on inflammatory signaling. (ii) Potential indirect effects on melanocytes: While GDF15 has been shown to stimulate melanogenesis in melanocytes through β-catenin/MITF/tyrosinase upregulation, this pro-pigmentary effect has been demonstrated primarily in the context of senescent fibroblast-derived GDF15. Whether the reduced GDF15 levels in psoriatic skin contribute to post-inflammatory pigmentary changes—and if so, through what mechanism—remains unknown and represents an important area for future investigation (3, 66).

In summary, rather than creating a GDF15-elevated state that links inflammation to pigmentation, the psoriatic inflammatory environment suppresses GDF15 expression via TNF-α/GATA2-dependent mechanism. This downregulation removes GDF15’s anti-inflammatory feedback, potentially perpetuating inflammation, while the link to melanocyte function—if it exists—would likely involve loss of GDF15’s paracrine effects on melanogenesis, a hypothesis that requires direct experimental validation (**Figure 1A**).

The key molecular mediators that connect these two domains are: (i) NF-κB and STAT3: In psoriatic skin, the inflammatory milieu is characterized by elevated levels of TNF-α, IL-17, and IL-22, which activate NF-κB and STAT3 signaling in keratinocytes and dermal immune cells. However, contrary to the expectation that these transcription factors would drive GDF15 upregulation, GDF15 expression is decreased in psoriatic epidermis. TNF-α suppresses GDF15 expression in keratinocytes by inhibiting the protein level of the transcription factor GATA2 (14). GDF15 itself functions as a negative regulator of inflammation by limiting keratinocyte cytokine and chemokine synthesis through inhibition of TAK1/NF-κB activation (14). Thus, rather than serving as upstream activators of GDF15 in psoriasis, NF-κB and STAT3 operate within an inflammatory milieu in which GDF15 is paradoxically suppressed, removing a critical anti-inflammatory brake. (ii) β-catenin: GDF15 stimulates melanogenesis in melanocytes through MITF/tyrosinase upregulation via β-catenin signaling (3). This stimulatory action has been confirmed in ex vivo cultured skin and in reconstituted human skin samples (3). GDF15 derived from senescent fibroblasts promotes skin pigmentation and may play a role in aging-associated pigmentation disorders such as senile lentigo and melasma (3). (iii) MITF and tyrosinase: These are melanocyte-specific transcription factors and enzymes that execute the pigmentary output downstream of GDF15/β-catenin signaling. GDF15 significantly upregulates the mRNA and protein expression levels of MITF and tyrosinase in human melanocytes (3).

This framework—linking inflammation to GDF15 dysregulation and GDF15 to melanocyte function via β-catenin/MITF/tyrosinase—provides a conceptual molecular basis for understanding pigmentary abnormalities observed in chronic inflammatory skin diseases, including psoriasis, atopic dermatitis, and post-inflammatory hyperpigmentation disorders (**Figure 1C**). However, it is important to note that the direct connection between inflammatory downregulation of GDF15 in psoriasis and subsequent pigmentary changes remains hypothetical; while GDF15’s pro-melanogenic effects are well established in the context of senescent fibroblasts (3), whether reduced GDF15 levels in psoriatic skin contribute to post-inflammatory pigmentary abnormalities—and if so, through what mechanism—has not been experimentally demonstrated and represents an important area for future investigation.

Another well-characterized cross-domain interaction connects dermal fibroblast senescence to epidermal melanocyte pigmentation. As described in Section 3, senescent fibroblasts—accumulating in aged and photoaged skin—secrete GDF15 as a core component of the senescence-associated secretory phenotype (SASP) (3). Kim et al. (3) demonstrated that GDF15 expression levels are increased in UV-irradiated senescent fibroblasts and photoaged hyperpigmented skin. This GDF15 acts on melanocytes via the β-catenin/MITF/tyrosinase pathway, driving age-associated hyperpigmentation (e.g., solar lentigines) (3, 28). The stimulatory action of GDF15 during pigmentation was further confirmed in ex vivo cultured skin and in a reconstituted human skin sample (3).

Bridging mediators: The key connection here is the paracrine SASP–GDF15–β-catenin–MITF axis (3, 28). Importantly, the upstream triggers for this axis—UV-induced p53 and ATF4/CHOP-mediated stress responses—are distinct from those in the inflammatory axis, demonstrating that different upstream stimuli converge on the same downstream effector (β-catenin/MITF) to produce similar pigmentary outcomes. GDF-15 is known to be regulated by p53 (28), and UV irradiation activates the integrated stress response involving ATF4 and CHOP (15).

Potential interactions with inflammation: In photoaged skin, low-grade chronic inflammation (“inflammaging”) may synergize with SASP-derived GDF15, potentially amplifying both the magnitude and duration of GDF15 elevation and thereby exacerbating pigmentary changes. Recent multi-system analysis of inflammatory solar lentigo has revealed upregulation of stress response-associated genes and inflammatory pathways in response to UV-induced oxidative stress, implying their involvement in the pathogenesis of inflammatory hyperpigmentation. The senescence of fibroblasts causes alteration of their secretory proteins, including GDF15, which has been identified as a key factor in solar lentigo pathogenesis (141). This intersection between aging-associated senescence and chronic inflammation—both GDF15 drivers—may explain the particularly prominent hyperpigmentation seen in photoaged skin with concurrent low-grade inflammation.

### 6.2 The inflammation–fibrosis axis: linking chronic inflammation to matrix remodeling

A third cross-domain interaction connects chronic cutaneous inflammation to fibrotic remodeling (exemplified by systemic sclerosis). In SSc, inflammatory infiltrates precede and likely drive fibrotic changes. GDF15, elevated in SSc and correlated with disease severity, may serve as one molecular link between these phases (28, 122, 123, 127).

Proposed mechanism: Inflammatory cytokines (TNF-α, IL-6) activate NF-κB and STAT3, driving GDF15 expression in fibroblasts and inflammatory cells (21, 46, 142) (**Figure 1C**). Elevated GDF15, in turn, modulates fibroblast collagen synthesis (*COL1A1*, *COL3A1*) through pathways that may involve SMAD2/3 or alternative signaling routes. Transcriptome analysis of bleomycin-induced SSc models has revealed significant upregulation of key pro-fibrotic genes including GDF15 in fibrotic skin and lung tissue, and mitochondrial dysfunction in SSc fibroblasts promotes the release of the mitokine GDF15. While the precise receptor mechanism remains unresolved (see Section 1.1.1), this connection places GDF15 at the interface between inflammatory initiation and fibrotic progression (**Figure 1B**).

Mediator genes: NF-κB, STAT3, SMAD2/3, COL1A1, and COL3A1 are key molecular nodes in this axis.

### 6.3 GDF15 as a multi-node hub: a conceptual framework

Collectively, the cross-domain interactions described above position GDF15 as a multi-node signaling hub that connects diverse cutaneous processes:

Key insights from this integrated framework: (i) Convergence: Different upstream stressors (inflammatory cytokines, UV, metabolic stress) converge on GDF15 upregulation. (ii) Divergence: Once elevated, GDF15 diverges to affect multiple downstream target cell types (melanocytes, fibroblasts, immune cells) through distinct effector pathways (β-catenin/MITF, SMAD/collagen, NF-κB feedback). (iii) Context-dependent outcomes: The net biological effect depends on which target cell type predominates, the duration of elevation, and the surrounding microenvironment. (iiii) Pathological amplification: In chronic disease states, GDF15 elevation may simultaneously affect multiple compartments (e.g., melanocytes and fibroblasts), amplifying tissue pathology.

### 6.4 Clinical and therapeutic implications of the integrated framework

This integrated perspective has several clinical implications: (i) Biomarker interpretation: Elevated GDF15 in psoriasis may not only reflect inflammatory activity but may also predict the development of post-inflammatory pigmentary changes. (ii) Therapeutic targeting: Interventions that normalize GDF15 may have pleiotropic benefits across multiple cutaneous domains simultaneously—reducing pigmentation, modulating inflammation, and limiting fibrosis. (iii) Patient stratification: The GDF15–β-catenin/MITF axis may identify patients at risk for pigmentation complications during anti-inflammatory therapy.

### 6.5 Evidence levels for proposed GDF15 mechanisms in skin

To assist readers in distinguishing experimentally validated findings from speculative hypotheses, we summarize the current evidence base for GDF15-mediated mechanisms in cutaneous biology using a simple evidence-level classification (**Table 5**).

**Table 5:** Evidence levels for proposed GDF15 mechanisms in skin.

Upstream domain	Stimulus/Signaling	GDF15 response	Downstream target domain	Key mediator genes	Pathological consequence	Ref.
Inflammation (Psoriasis)	TNF-α → GATA2 inhibition	GDF15 ↓	Inflammation (feedback loss)	GATA2 → GDF15 → TAK1/NF-κB, Rap1	Loss of anti-inflammatory brake, neutrophil infiltration	(14)
Inflammation (Psoriasis)	Hypothetical: TNF-α/IL-17 → NF-κB/STAT3	GDF15 ↓ (paradoxical)	Pigmentation (Melanocytes)	Unknown (GDF15 → β-catenin/MITF/tyrosinase established in senescence context)	Post-inflammatory pigmentary changes (hypothetical)	(3, 14)
Senescence (Photoaging)	UV → p53	GDF15 ↑ (SASP)	Pigmentation (Melanocytes)	p53 → GDF15 → β-catenin, MITF, tyrosinase	Age-associated hyperpigmentation (solar lentigines)	(3, 28)
Inflammation/Fibrosis (SSc)	Bleomycin/TGF-β pathway activation	GDF15 ↑	Fibrosis (Fibroblasts)	GDF15 → SMAD2/3, COL1A1, COL3A1; IL-6, CCL2	Dermal fibrosis, ECM accumulation	(40, 46, 122, 123)

However, these implications remain hypothetical and require validation through longitudinal studies and interventional trials. The integrated framework is intended to generate testable hypotheses rather than to provide definitive clinical guidance.

## 7 Future target development and prospects

### 7.1 Technical approaches for developing GDF15 targets

Interest in GDF15 as a therapeutic target for skin diseases has emerged from observations of its involvement in pigmentation, aging, and immune regulation. Translating these observations into therapeutic applications faces significant methodological and validation challenges (15, 28, 48) (**Figure 1D**).

Recent advances in structural biology, signaling pathway mapping, and drug discovery technologies have provided conceptual frameworks for exploring GDF15 modulation. However, direct dermatological applications remain at an early, exploratory stage (143).

#### 7.1.1 Screening strategies for small molecule modulators of GDF15 signaling

Identifying small molecules that modulate GDF15 signaling represents a crucial first step in therapeutic development. High-throughput screening (HTS) platforms have been proposed to identify compounds that interact with GDF15 or its downstream effectors. Structure-based drug design, using three-dimensional modeling of the GDF15-GFRAL receptor complex, has shown promise in other disease areas and may help identify binding sites relevant to skin biology, though these approaches require independent validation for cutaneous applications (144, 145).

Virtual screening combined with molecular docking can filter large compound libraries, providing starting points for laboratory evaluation. Pathway-centered assays measuring apoptosis, inflammation, and oxidative stress offer functional readouts to assess compound effects (134, 143). These strategies have proven effective in systems involving MITF, CREB, and Wnt/β-catenin signaling-pathways relevant to pigmentation and photoaging. Applying similar tools to GDF15 pathways may reveal new modulators, though extensive experimental confirmation is essential (146, 147).

Three-dimensional skin models containing melanocytes, fibroblasts, and keratinocytes provide a more physiologically relevant context for evaluating candidate compounds. These models can simulate conditions such as UV-induced pigmentation or fibroblast senescence, offering insights into tissue-specific drug effects. While their predictive value awaits validation, such models may bridge early discovery and preclinical development (3, 15, 148, 149).

#### 7.1.2 Exploratory approaches to GDF15 modulation: inhibitors, agonists, and emerging concepts

Three major strategies for modulating GDF15 activity have been proposed based on recent pharmacological reviews, though all remain in preclinical or early experimental stages.

GDF15 neutralizing antibodies are being investigated as potential inhibitors. These antibodies are designed to bind GDF15 and prevent its interaction with receptors such as GFRAL, potentially limiting pathological GDF15 signaling in diseases where overactivation contributes to inflammation or fibrosis. Early laboratory studies have demonstrated feasibility, but no dermatology-specific clinical data are available (45, 150–152).

Small molecule inhibitors targeting either GDF15 itself or its receptor interactions represent another exploratory avenue. While initial compounds have shown activity in non-cutaneous models, their selectivity, stability, and skin-specific safety profiles remain uncharacterized. Combination with anti-TGF-β or cytokine-targeting biologics has been hypothesized to produce additive effects, though this remains speculative and requires careful experimental evaluation, The in silico screening has proven effective in identifying small organic molecules (SOMs) putatively capable of inhibiting GDF15, among which SOM D appears the most promising. Further characterizations will be necessary to better understand the exact mechanisms of action.” (153). Moreover, NGM120 is an antibody designed to bind to the receptor (known as GFRAL) for GDF15. This binding blocks the interaction of GDF15 with GFRAL, which, in turn, inhibits the activity of GDF15, potentially alleviating hyperemesis gravidarum (HG) symptoms (https://www.ngmbio.com/news/release/ngm-bio-announces-first-participant-dosed-emerald-phase-2).

GDF15 agonists or analogs are being explored for their potential to enhance protective or reparative functions, such as tissue regeneration and pigment regulation (154). In mouse and human lungs with interstitial fibrosis, CST3 and GDF15 expressions were markedly reduced, and the restoration of these cytokines alleviated the fibrotic changes in mouse lungs (154). Recombinant long-acting GDF15 analogs and synthetic peptides mimicking active domains are of particular interest in pigmentary disorders and inflammatory skin diseases. These molecules could theoretically activate beneficial GDF15 pathways, but they remain in proof-of-concept phases with no confirmed therapeutic efficacy.

The choice between GDF15 inhibition or activation may depend on underlying disease mechanisms. Inhibition could be prioritized in chronic inflammatory conditions such as psoriasis, whereas activation might be considered for pigmentary loss disorders or tissue repair contexts. In systemic sclerosis, indirect modulation of GDF15 within the SASP-TGF-β axis may be most relevant during early, reversible disease stages. These are conceptual models rather than clinical recommendations and require validation through rigorous preclinical studies.

Current efforts face significant challenges, including ensuring molecular specificity, achieving skin-selective delivery, and avoiding systemic metabolic effects of GDF15 modulation. At present, all GDF15-targeting strategies remain experimental, with no confirmed dermatological clinical trial data.

### 7.2 Challenges and strategies for clinical translation of GDF15

#### 7.2.1 Key gaps and future research directions

Translating GDF15-related discoveries into clinical applications faces several obstacles. The understanding of GDF15’s interactions with established signaling networks remains incomplete. Pathways such as TGF-β, Wnt/β-catenin, and CREB are well studied in skin biology, yet their interplay with GDF15 is not fully delineated. Clarifying these relationships will be essential for predicting therapeutic outcomes and avoiding unintended effects (3, 40, 155–158).

Emerging evidence suggests additional regulatory axes may be involved. Voltage-gated sodium channels (Nav1.7/1.8) and cholinergic signaling pathways have been proposed as potential modulators of GDF15-related processes in tissue remodeling. However, direct evidence in human skin cells is currently limited, highlighting the need for prospective mechanistic studies (159–162).

Variability in experimental outcomes likely reflects genetic, epigenetic, and environmental heterogeneity. Advanced systems biology and computational approaches could help map this complexity and identify consistent patterns. Preclinical models that more accurately represent human skin physiology will be critical for bridging discovery-stage findings to translational research (48, 163, 164).

Future studies should integrate multi-omics platforms-transcriptomics, proteomics, and metabolomics-to create comprehensive maps of GDF15-related signaling under both physiological and pathological conditions. Live-cell and single-cell imaging techniques may provide real-time insights into GDF15 dynamics within the skin microenvironment. Large-scale collaborative research efforts will be needed to generate reproducible datasets and inform rational therapeutic development (48, 163).

#### 7.2.2 Multidisciplinary collaboration for GDF15 applications

Advancing GDF15-based interventions requires integration across dermatology, molecular biology, pharmacology, and computational sciences. Dermatology provides clinical context, particularly in conditions such as pigmentary disorders, systemic sclerosis, and autoimmune skin diseases. Molecular biology contributes insights into cross-pathway regulation, including GDF15’s interactions with MITF, CREB, and the IL-23/Th17 immune axis (**Figure 2B**) (3, 28, 110, 158, 165, 166).

Drug delivery innovations, such as nanoparticle carriers and transdermal formulations, may enhance tissue specificity and reduce systemic exposure of GDF15 modulators from a pharmacological standpoint (167–169). Computational biology offers tools for analyzing complex datasets and predicting interactions. Machine learning models may help identify biomarkers of response or potential co-targets, though their predictive validity awaits confirmation (170, 171).

Collaborative networks combining these disciplines can create robust experimental pipelines from discovery to translational research. While the potential is considerable, current progress remains exploratory (72, 172–174). No GDF15-based therapeutic strategy for dermatological conditions has demonstrated confirmed clinical efficacy, underscoring the importance of cautious, stepwise validation.

### 7.3 Critical analysis of the context-dependent functions of GDF15 in skin

A recurring theme throughout this review is the context-dependent nature of GDF15 function—its ability to exert seemingly opposing effects depending on the biological setting. While we have invoked this concept throughout the preceding sections, a critical reappraisal is warranted to assess the robustness of these apparent contradictions and to identify what is genuinely known versus what remains inferential. Below, we critically examine two central dichotomies: pro-inflammatory versus anti-inflammatory and pro-aging versus anti-aging activities of GDF15 (**Figure 2C**).

#### 7.3.1 Pro-inflammatory versus anti-inflammatory: a true dichotomy or an artifact of context?

The literature presents GDF15 as both a promoter and a suppressor of inflammation. How should these opposing reports be interpreted?

Evidence for anti-inflammatory actions. The most compelling evidence for an inflammation-limiting role of GDF15 comes from murine genetic studies. In the imiquimod-induced psoriasis model, GDF15-knockout mice exhibited exacerbated inflammation with increased neutrophil infiltration and keratinocyte hyperproliferation, accompanied by attenuated TAK1/NF-κB activation (14). Similarly, in autoimmune models of SLE, GDF15 deficiency worsened lymphoproliferation and humoral responses (4, 64, 117). These data establish that endogenous GDF15 exerts a protective, inflammation-limiting function in certain acute and autoimmune settings.

Evidence for pro-inflammatory actions. By contrast, human correlative studies consistently show that elevated circulating GDF15 associates with inflammatory disease severity, including psoriasis, SLE, and metabolic syndrome (71, 115). GDF15 has also been shown to promote type 2 inflammatory responses in some contexts (64).

Critical assessment: Several factors complicate direct comparison of these findings: (i) Genetic deficiency (knockout) versus elevated levels are not symmetric comparisons. GDF15-knockout studies address the loss of a basal protective function, whereas elevated GDF15 in human disease reflects gain or dysregulation of the system. These are distinct biological questions that need not yield opposite answers. A protective protein can be elevated as a compensatory response to inflammation—its elevation does not negate its protective role but rather indicates an attempted homeostatic response. (ii) Acute versus chronic exposure likely matters fundamentally. In the acute imiquimod model, GDF15 deficiency worsens disease—consistent with a protective role early in the inflammatory response. In chronic human disease, sustained GDF15 elevation may reflect prolonged stress signaling that, over time, contributes to maladaptive outcomes (metabolic dysregulation, fibrosis) even if the initial function was protective. (iii) Species differences and disease models must be acknowledged. The imiquimod model—while widely used—does not fully recapitulate human psoriasis, which involves complex genetic, environmental, and immune interactions spanning years or decades (**Figure 2C**).

Critical conclusion: The apparent pro- versus anti-inflammatory dichotomy may be more apparent than real. A more parsimonious model is that GDF15 is fundamentally a stress-responsive homeostatic factor whose net effect depends on the timing, duration, and systemic context of its elevation. Anti-inflammatory effects are most evident in acute, genetically deficient states; pro-inflammatory associations are most evident in chronic, correlative human studies. Whether these represent two faces of the same molecule or distinct phenomena requiring separate mechanistic explanations remains an open question.

#### 7.3.2 Pro-aging versus anti-aging: stress protection versus chronic deterioration

A similar duality characterizes GDF15’s role in aging. GDF15 has been described as both protective against age-related stress and a driver of age-associated pathology (**Figure 2C**).

Evidence for anti-aging/protective functions. GDF15 is induced by multiple forms of cellular stress (UV, oxidative stress, ER stress) and appears to promote cell survival under acute stress conditions (27, 175–177). In the skin, acute UV-induced GDF15 may activate DNA repair mechanisms and limit apoptosis, suggesting a cytoprotective function. In cardiovascular and metabolic systems, GDF15 is protective against ischemia-reperfusion injury and metabolic stress.

Evidence for pro-aging/deleterious functions. Senescent fibroblasts secrete GDF15 as a core SASP component (3), and chronic GDF15 elevation is associated with frailty, sarcopenia, and age-related metabolic decline. In the skin, chronic GDF15 from senescent fibroblasts drives age-associated hyperpigmentation via β-catenin/MITF activation (3). These findings position GDF15 as a mediator of the deleterious effects of cellular senescence.

Critical assessment. The distinction between acute stress protection and chronic maladaptive signaling appears central. In the acute setting, GDF15 functions as a stress sensor and survival factor—its induction helps cells cope with transient challenges. In the chronic setting, persistent GDF15 elevation—particularly from senescent cells—contributes to the paracrine spread of senescence-associated phenotypes, driving the very age-related changes that characterize the aging skin (**Figure 2A**).

Key unanswered questions include: (i) At what threshold does GDF15 transition from protective to pathological? (ii) Can the protective acute effects be preserved while limiting chronic deleterious signaling? (iii) Is GDF15 a driver of skin aging or a consequence of the aging process? The current evidence is predominantly correlative, and causality has not been rigorously established in human skin aging.

Critical conclusion: Similar to its inflammatory roles, GDF15’s aging-related functions are likely phase-dependent rather than intrinsically dichotomous. The molecule itself is neither “pro-aging” nor “anti-aging”; rather, its biological outcome is dictated by the duration, magnitude, and cellular source of its expression, as well as the broader tissue microenvironment.

### 7.4 General lessons and future directions

Several overarching principles emerge from this critical analysis: (i) Evidence quality varies considerably. Murine genetic studies provide strong evidence for causation in specific contexts; human correlative studies provide evidence for association but not causation. These should not be conflated (122, 123). (ii) Publication bias cannot be excluded. It is plausible that anti-inflammatory and anti-aging effects are more likely to be published because they are mechanistically interesting and conceptually appealing, potentially skewing the perceived balance of evidence (3, 40). (iii) Context-dependency is a useful framework but not a satisfactory explanation. Future studies must move beyond invoking “context-dependency” as a *post-hoc* explanation and instead identify the specific molecular switches that determine outcome—whether these are receptor isoforms, signaling thresholds, or microenvironmental co-factors (14, 172). (iiii) Temporal dynamics are underexplored. Most studies examine single time points. Understanding how GDF15 levels and functions change over the course of disease progression or aging is essential for distinguishing cause from consequence (5, 178). (iiiii) Therapeutic implications are uncertain. The dual nature of GDF15 poses a fundamental challenge for therapeutic targeting. Enhancing GDF15 may benefit acute inflammatory conditions but could exacerbate chronic, senescence-associated pathology. Conversely, inhibiting GDF15 may alleviate chronic pathology but could impair acute stress responses. Phase-specific and cell-type-specific targeting strategies—if feasible—would be required to realize therapeutic potential while minimizing unintended consequences (179–181).

### 7.5 Therapeutic implications: challenges and limitations

The preceding discussion of GDF15’s pleiotropic roles in skin biology naturally raises the question of whether GDF15 pathways represent viable therapeutic targets. While this possibility is conceptually appealing, several fundamental challenges must be acknowledged, and current evidence does not yet support the development of GDF15-directed therapies for skin diseases.

Challenge 1: The receptor conundrum. As discussed in Section 1.1.1, the receptor(s) mediating GDF15’s cutaneous effects remain unidentified. Unlike the well-characterized GFRAL–RET axis in the brainstem, which mediates systemic metabolic effects (34, 182), the receptor system responsible for GDF15 actions in melanocytes, fibroblasts, and keratinocytes is unknown (40). GFRAL expression is limited to hindbrain neurons and is not present in peripheral tissues (34). Although recent studies have identified GDF15 interactions with TGFBR2 and GFRAL on melanoma cells (126), whether these receptors mediate GDF15’s effects in normal cutaneous cell types—melanocytes, fibroblasts, and keratinocytes—remains unresolved. Without a defined receptor target, rational drug design—whether small molecule agonists/antagonists or biologics—is severely hampered. This is not a minor technical gap but a fundamental obstacle to therapeutic development (40).

Challenge 2: The duality problem. GDF15 exhibits opposing effects depending on context: protective in some settings (acute inflammation, stress responses), pathogenic in others (chronic inflammation, senescence-associated pathology) (50, 183). It may act as a protective response by limiting inflammation and cellular injury; yet, in other contexts, it contributes to fibrogenesis, tumor progression, immunosuppression, and cachexia (183). In organ fibrosis, GDF15 can either contribute to fibrosis by promoting inflammation and fibroblast activation or confer protective effects by modulating the immune response and mitigating fibrosis severity, with the exact role varying depending on the organ involved and the specific disease context (40). This duality poses a significant therapeutic risk: enhancing GDF15 activity could alleviate one condition while exacerbating another. For example, GDF15 augmentation might benefit acute psoriatic flares (based on murine protection data) but could worsen chronic pigmentation or fibrotic complications. Conversely, GDF15 inhibition might reduce hyperpigmentation but impair protective stress responses (51).

Challenge 3: Causal versus correlative uncertainty. The majority of human evidence linking GDF15 to disease severity is cross-sectional and correlative. Whether GDF15 elevation drives pathogenesis or represents an epiphenomenon of disease activity remains unclear (184). Human population cohort data do not support a role for moderately elevated GDF15 concentrations as a causal factor in cardiometabolic disease but support its role as a biomarker of metabolic stress (8). Similarly, in atherosclerosis, GDF15 in circulation is a potential biomarker, but not a causal mediator, of atherosclerosis (185). A factor can be an excellent biomarker (reflecting disease activity) while having no causal role—and therefore no therapeutic utility as a direct target. This distinction is critical and often overlooked in the enthusiasm for “targetable” molecules (40).

Challenge 4: Target specificity and delivery. Even if a therapeutic strategy targeting GDF15 or its receptors were developed, achieving tissue-specific modulation would be essential to avoid systemic side effects. Systemic GDF15 modulation would disrupt its well-established metabolic functions in appetite regulation and energy homeostasis via the brainstem GFRAL–RET axis (34, 182), potentially causing unintended weight changes or metabolic disturbances. Limited tissue specificity restricts the diagnostic value of GDF15, and tailored treatment strategies remain speculative (180). The technology for selective cutaneous delivery of GDF15-modulating agents—whether by topical formulations, intradermal injection, or other approaches—remains in its infancy, with transdermal delivery of macromolecules facing substantial barriers due to the stratum corneum effectively inhibiting macromolecular passage (186).

Current state of evidence for therapeutic strategies. To provide clarity, we summarize the evidence stage for each proposed therapeutic approach in (**Table 6**). Critical conclusion. At present, the therapeutic implications of GDF15 in dermatology are conceptual and aspirational rather than clinically actionable. The primary value of the current evidence base is in generating testable hypotheses and identifying knowledge gaps—not in providing immediate therapeutic direction. The most productive near-term application of GDF15 research may be in biomarker development (monitoring disease activity and metabolic comorbidities) rather than in therapeutic targeting.

**Table 6:** The evidence stage for each proposed therapeutic approach of GDF15 in skin.

Therapeutic strategy	Examples	Current evidence stage	Major obstacle	Ref.
Neutralizing anti-GDF15 antibodies	To reduce chronic GDF15 elevation	Preclinical only; no skin-specific data	Unknown cutaneous receptor; risk of blocking protective functions	(40, 114, 187)
GDF15 receptor agonists/antagonists	To enhance or block receptor signaling	Conceptual; no skin-targeted agents	Receptor unknown in skin	(17, 126, 187)
Small molecule modulators of GDF15 expression	Targeting upstream regulators (p53, NF-κB, ATF4)	Indirect; not GDF15-specific	Lack of specificity; pleiotropic effects of upstream targets	(14, 28, 188, 189)
siRNA/antisense targeting GDF15	To reduce local GDF15 production	Preclinical concept	Delivery challenges; unclear therapeutic benefit	(186)
GDF15-based diagnostics	Biomarker development	Clinical correlation established	Requires validation; specificity issue	(44, 122, 123, 190)

We emphasize this not to discourage investigation but to provide a balanced and scientifically honest assessment of where the field currently stands. Until the fundamental questions—receptor identity, causal role, and context-specific mechanisms—are resolved, therapeutic claims must remain appropriately circumscribed.

## 8 Conclusion

### 8.1 Summary of the key roles of GDF15 in skin diseases

#### 8.1.1 Core position of GDF15 in pigmentation biology, aging regulation, and immune networks

Across skin biology, GDF15 operates as an integrative node coupling environmental stress sensing with cellular programs in melanocytes, fibroblasts, and immune cells (28). In pigmentation, convergent evidence supports a model in which GDF15 coordinates melanocyte responses to oxidative and inflammatory cues, thereby fine-tuning melanogenesis under both physiological and pathological conditions (3). This coordination is most likely mediated through ROS-linked signaling and paracrine crosstalk between dermal fibroblasts and melanocytes within TGF-β-rich microenvironments, positioning GDF15 as a regulator rather than a passive component of pigment homeostasis.

In aging skin, GDF15 sits at the intersection of redox balance, matrix remodeling, and chronic low-grade inflammation [5.15.106]. The emerging view is phase-dependent: a transient GDF15 pulse helps resolve acute stress, but when levels remain high-as in chronically sun-damaged dermis-the balance tips toward collagen breakdown and uneven pigmentation (**Figure 2A**).

Within immune-mediated dermatoses, GDF15 interfaces with the IL-23/Th17-IL-17 network and related T-cell circuits (**Figure 2B**). In psoriasis, available data remain contradictory: several studies report serum and lesional elevation correlated with disease activity, whereas others describe reduced expression in plaques (14, 71). This heterogeneity indicates that the precise role of GDF15 in psoriatic inflammation remains unresolved. In SLE, evidence more consistently points to a biphasic pattern, with acute rises corresponding to compensatory regulation and lower levels during remission (109–111). These patterns indicate that the effects of GDF15 are concentration- and context-dependent, and that timing relative to disease phase is a primary determinant of outcomes (**Table 4**; **Figure 2C**).

#### 8.1.2 Integrative value for dermatology

At the level of clinical reasoning, GDF15 offers two immediate utilities without presupposing specific interventions. First, as a cross-axis signal linking oxidative stress, fibrosis, and adaptive immunity, it helps reconcile apparently opposite observations across pigmentation, aging, and autoimmunity by providing a single, tunable control point. Second, its dynamic behavior across disease phases suggests a principled way to read skin pathology temporally: early, adaptive GDF15 pulses versus late, persistent elevation (3, 28).

### 8.2 Limitations of the present review

This is a narrative rather than a systematic synthesis, and the selection of literature was not protocol-driven; important studies may have been missed, and no formal study-quality appraisal was performed. The bidirectional, threshold-like behavior of GDF15 complicates definitive statements, particularly when experimental systems differ in cell types (133), exposure duration, and readouts. Much of the evidence comes from *in vitro* or animal work, with sparse longitudinal human data establishing temporal causality (191). Publication bias cannot be excluded. Assay heterogeneity and lack of standardized reference ranges limit cross-study comparability (192). Finally, several mechanistic breakpoints—such as the concentration ranges separating adaptive from pathogenic responses, or the proposed TGF-β:GDF15 ratio as a stage indicator-remain theoretical and require prospective validation before clinical use (133, 193) (**Figure 2C**).

## Glossary

AMPK: AMP-activated protein kinase
ATF4: Activating transcription factor 4
CCL20: C-C motif chemokine ligand 20
CHOP: C/EBP homologous protein
COL1A1: Collagen type I alpha 1 chain
COL3A1: Collagen type III alpha 1 chain
CREB: cAMP response element-binding protein
CRISPR-Cas9: Clustered regularly interspaced short palindromic repeats-associated protein 9
ECM: Extracellular matrix
EGR1: Early growth response protein 1
ERK: Extracellular signal-regulated kinase
GATA2: GATA binding protein 2
GDF15: Growth differentiation factor 15
GFRAL: GDNF family receptor alpha-like
HF: Heart failure
HTS: High-throughput screening
IL: Interleukin
ISR: Integrated stress response
MAPK: Mitogen-activated protein kinase
MIC-1: Macrophage inhibitory cytokine 1
MITF: Microphthalmia-associated transcription factor
MMP: Matrix metalloproteinase
NF-κB: Nuclear factor kappa-light-chain-enhancer of activated B cells
PASI: Psoriasis Area and Severity Index
PKA: Protein kinase A
ROS: Reactive oxygen species
SANRA: Scale for the Assessment of Narrative Review Articles
SASP: Senescence-associated secretory phenotype
SCF: Stem cell factor
SLE: Systemic lupus erythematosus
SMAD: Mothers against decapentaplegic homolog
SSc: Systemic sclerosis
STAT3: Signal transducer and activator of transcription 3
TAK1: Transforming growth factor-β-activated kinase 1
TGF-β: Transforming growth factor-beta
Th17: T helper 17 cell
TNF-α: Tumor necrosis factor-alpha
TYR: Tyrosinase
TYRP1: Tyrosinase-related protein 1
TYRP2: Tyrosinase-related protein 2
UV: Ultraviolet.
